# Families caring for children and adolescents with rare disease: a health economic evaluation of face-to-face and online psychosocial support intervention of the randomized controlled CARE-FAM-NET study

**DOI:** 10.1186/s13561-026-00883-0

**Published:** 2026-10-26

**Authors:** Nicolas Pardey, Kristine Kreis, Anna Leidger, Johannes Boettcher, Jonas Denecke, Sara Soeiro Gomes, Ania C. Muntau, Anne Daubmann, Ann-Kathrin Ozga, Antonia Zapf, Karl Wegscheider, Miriam Rassenhofer, Jörg Fegert, Dunja Tutus, Ursula Marschall, Stefanie Schellhammer, Sophia Rocabado, Thomas Krull, Anja Wadeck, Udo Schneider, Steve Hüttmann, Michél Henkel, Johann-Matthias von der Schulenburg, Silke Wiegand-Grefe, Jan Zeidler

**Affiliations:** 1 LiKe Healthcare Research GmbH, Berlin, Germany; 2 Leibniz University Hannover, Center for Health Economics Research Hannover (CHERH), Königsworther Platz 1, 30167 Hannover, Germany; 3 Department of Psychiatry and Psychotherapy, University Medical Center Hamburg-Eppendorf, Hamburg, Germany; 4 Department of Child and Adolescent Psychiatry, Psychotherapy and Psychosomatics, University Medical Center Hamburg-Eppendorf, Hamburg, Germany; 5 University Children’s Hospital, University Medical Center Hamburg-Eppendorf, Hamburg, Germany; 6 Institute of Medical Biometry and Epidemiology, University Medical Center Hamburg-Eppendorf, Hamburg, Germany; 7 Child and Adolescent Psychiatry, Psychosomatics and Psychotherapy, Ulm University Hospital, Ulm, Germany; 8 Barmer, Statutory Health Insurance, Wuppertal, Germany; 9 DAK-Gesundheit, Statutory Health Insurance, Hamburg, Germany; 10 Mobil Krankenkasse, Statutory Health Insurance, Hannover, Germany; 11 Techniker Krankenkasse, Statutory Health Insurance, Hamburg, Germany; 12 IKK Classic, Statutory Health Insurance, Dresden, Germany; 13 Kaufmännische Krankenkasse (KKH), Statutory Health Insurance, Hannover, Germany; 14 German Center for Child and Adolescent Health, Hamburg, Germany

**Keywords:** ICER, Psychosocial intervention, Rare disease, Caregiver, Claims data, Health economic evaluation

## Abstract

**Background:**

Parents caring for children with rare diseases often experience a reduced Health-Related Quality of Life (HRQoL). This study addresses the growing need for therapeutic support by evaluating two innovative forms of care: CARE-FAM (CF) and a web-based program (WEP) WEP-CARE (WC). CF is a psychodynamic-psychosocial intervention with eight sessions per family over six months, aiming to improve mental health, HRQoL, and satisfaction of all family members. WC is an online intervention leveraging cognitive-behavioral writing therapy principles to achieve the same objectives. The aim of this study was to evaluate the incremental cost per quality-adjusted life year (QALY) gained for CF, WC and their combination (CF + WC) compared with the control group (CG), on a per-parent basis, over an 18-month time horizon from the German statutory health insurance (SHI) payer perspective.

**Methods:**

Children Affected by Rare disease and Their Families – Research Network (CARE-FAM-Net) is a 2 × 2 factorial, multicenter randomized controlled study conducted across 17 psychiatric hospitals in Germany with three intervention groups (CF, WC, CF + WC) and a control group. Cost evaluations utilized claims data from seven SHI funds, reflecting the SHI payer perspective, while QALYs were calculated using the EQ-5D-3 L score at four points over an 18-month time horizon. The unit of analysis was the individual parent, therefore all costs and QALYs were estimated on a per-parent basis. The primary analysis used the intention-to-treat statutory health insurance (ITT-SHI) population. Missing data were addressed through multiple imputation by chained equations with predictive mean matching, generating 20 imputed datasets that were combined using Rubin’s rules. Within-family correlation was accounted for in the statistical analyses. Numerical incremental cost-effectiveness ratios (ICERs) were reported where appropriate, and non-parametric family-cluster bootstrapping was performed to estimate statistical uncertainty. The sensitivity analysis used a restricted population. Costs and QALYs were discounted at a rate of 3%.

**Results:**

The primary ITT-SHI analysis included 653 participants, with a mean age of 39.4 years (SD 7.2), and 396 (60.6%) were female. Unadjusted mean annual discounted costs per parent were €4476 (SE €631) for CG, €3539 (SE €755) for CF, €3970 (SE €1249) for WC and €5518 (SE €1850) for CF + WC. Unadjusted mean annual discounted QALYs per parent were 0.9144 (SE 0.0079) for CG, 0.9215 (SE 0.0097) for CF, 0.9287 (SE 0.0074) for WC and 0.9313 (SE 0.0075) for CF + WC. Based on the incremental mean costs and baseline-adjusted QALY differences, CF and WC dominated CG, whereas CF + WC had an ICER of €109,371 per QALY gained. However, none of the between-group differences in costs or baseline-adjusted QALYs were statistically significant. At a willingness-to-pay (WTP) of €50,000 per QALY, the probabilities of cost-effectiveness in pairwise comparisons with CG were 84.2% for CF, 79.9% for WC, and 43.0% for CF + WC. When all strategies were compared simultaneously, WC had the highest probability of being the most cost-effective strategy (48.2%), followed by CF (37.6%), CF + WC (10.4%) and CG (3.8%). The sensitivity analysis yielded comparable results for CF and WC, whereas CF + WC also dominated CG. Leave-one-out analyses demonstrated sensitivity to individual extreme cost observations.

**Conclusions:**

In the primary analysis, CF and WC were associated with lower mean costs and positive baseline-adjusted QALY differences relative to CG, whereas CF + WC was associated with higher mean costs and a positive baseline-adjusted QALY difference. None of the differences in costs or baseline-adjusted QALYs were statistically significant. The sensitivity analysis yielded broadly comparable findings for CF and WC, whereas results differed for CF + WC. Given the study limitations, particularly the relatively small sample size, the current evidence is insufficient to support a definitive recommendation for implementation into routine care from a health economic perspective.

**Trial registration:**

The study was registered at 18th December 2018 at the German clinical trials register (Deutsches Register Klinischer Studien, DRKS, nr: DRKS00015859).

**Supplementary Information:**

The online version contains supplementary material available at https://doi.org/10.1186/s13561-026-00883-0.

## Introduction

According to the European Union’s definition, a disease is classified as rare if it affects no more than 5 in 10,000 individuals [1]. Over 6000 rare diseases impact between 18 and 30 million people within the European Union [2, 3]. Approximately 70–80% of these rare diseases are genetic, often affecting children [3, 4].

Numerous studies have investigated the Health-Related Quality of Life (HRQoL) of parents caring for children with rare diseases [5–8]. A recent systematic review highlighted that these parents experience reduced HRQoL compared to parents of healthy children and the general population [8]. While most studies found no impact on the physical domain, significant reductions in the psychosocial domain of HRQoL were reported. Mori et al. (2017) demonstrated that mothers caring for individuals with CDKL5 deficiency disorders, a rare genetic condition causing epileptic encephalopathy, exhibited lower HRQoL than the general population. Although physical HRQoL was not significantly different, their mental Short-Form 12 (SF-12) health survey score was 41.9 (standard deviation (SD) 11.6), revealing reduced mental HRQoL [6]. Bourke et al. (2008) found that mothers caring for children with Down syndrome had significantly lower HRQoL compared to Australian population norms, with a score of 45.2 (SD 10.6) on the SF-12 [7]. Witt et al. (2019) also reported diminished mental HRQoL for parents caring for children with achondroplasia, a rare skeletal dysplasia, without significant differences in physical HRQoL compared to the German reference population [5]. Additionally, both self-reported and parent-reported HRQoL for children with rare diseases were negatively affected compared with German population norms [9].

The reduced HRQoL experienced by parents of children with rare diseases has led to a growing demand for psychotherapeutic interventions. To address this need, the randomized controlled multicenter study “Children Affected by Rare disease and Their Families – Research Network” (CARE-FAM-NET) was conducted, providing two innovative forms of care: CARE-FAM (CF) and WEP-CARE (WC). CF is a face-to-face, low-frequency, family-oriented intervention designed to enhance the mental health, HRQoL and satisfaction of all family members. During the COVID-19 pandemic, face-to-face sessions were conducted online. In contrast, WC is an online intervention based on cognitive-behavioral writing therapy, tailored for severely distressed parents of chronically ill children [10]. Both interventions are new care models in Germany that have not yet been evaluated from a health-economic perspective.

The primary outcome of the main clinical study was a binary outcome indicating whether at least one parent within a family had a mental health diagnosis at 18 months post-baseline. Mental health diagnoses were assessed using the “Structured Clinical Interview for DSM-IV Axis I Disorders” (SCID-I) by independent, trained raters external to the project who were blinded to group allocation. No statistically significant differences in this binary outcome were observed between families receiving the respective intervention and those not receiving it at 18 months post-baseline.

This study aims to conduct a health economic evaluation of CF, WC and their combination (CF + WC), focusing on parental costs and quality-adjusted life years (QALYs). Although the interventions did not demonstrate superiority in terms of mental health diagnoses, the health economic analysis remains of significant interest. First, multiple factors beyond mental health diagnoses contribute to quality of life and the resulting QALYs. Second, the health economic analysis included all parents enrolled in the primary randomized controlled trial (RCT) for whom statutory health insurance (SHI) claims data were available, regardless of whether they had a mental health diagnosis. Consequently, it captures potential changes in health-related quality of life beyond clinically diagnosed mental disorders. Although family-oriented interventions may also affect children, the present health economic evaluation focused on parental costs and outcomes to ensure comparability across all intervention groups, particularly because WC was delivered exclusively to one parent. Child-specific outcomes and healthcare costs were therefore not included in the present analysis.

The evaluation results will assist healthcare system decision-makers in determining whether these innovative forms of care should be integrated into standard care.

## Methods

### Study design and sample

CARE-FAM-Net is a 2 × 2 factorial randomized controlled multicenter study conducted across 17 psychiatric hospitals in Germany [10]. The study period was 18 months. Data were collected at baseline (T1, prior to randomization), 6 (T2), 12 (T3), and 18 months (T4) after baseline. Comprehensive details on the participating psychiatric centers, sample size calculation, randomization, interventions, and further relevant information are documented in the study protocol [10].

The analysis is based on primary data and claims data from the participating parents. Claims data were obtained from participating SHI funds. The study included seven SHI funds, collectively encompassing 41% of the German market share. Claims data provided insights into service utilization and associated costs, covering outpatient and inpatient services, institutional outpatient care, prescribed drugs, remedial therapies, rehabilitation, and disability and sickness benefits over the 2018–2022 period. The primary data relevant for this cost-utility analysis included demographics (age, gender) and HRQoL assessed at each measurement point via the EQ-5D-3 L questionnaire [11].

Eligibility criteria required families to have at least one child aged 0–21 years with a suspected or confirmed rare disease, proficient German language skills among family members, and initial coverage from participating SHI funds. Exclusion applied to families manifesting severe psychiatric disorders with acute symptoms, including suicidal tendencies, massive self-injurious behavior, or acute psychotic symptoms [10]. Due to low enrollment during the study, the study team removed the eligibility criterion requiring insurance with a participating SHI fund. Consequently, we had no SHI claims data and healthcare cost information for participants insured with non-participating funds and therefore excluded them from the health economic analysis. The primary health economic analysis used the intention-to-treat SHI (ITT-SHI) population, comprising all randomized participants in the health economic evaluation population with available SHI claims data. This included participants with no observed post-baseline EQ-5D-3 L assessments as well as those with one or more observed post-baseline assessments. We restricted the sensitivity analysis to participants with at least two observed EQ-5D-3 L assessments across the four measurement points (T1-T4).

In compliance with the Consolidated Health Economic Evaluation Reporting Standards (CHEERS) [12], our study methods and results adhere to high reporting standards for health economic evaluations (Supplementary Table 1). Data were collected as part of the CARE-FAM-NET study [10].

### Randomization

The Institute of Medical Biometry and Epidemiology of the University Medical Center Hamburg-Eppendorf prepared computerized lists for family-wise randomization with variable block length, stratified by recruitment centers. According to a 2 × 2 factorial study design, each family was randomized to one of four combinations of the two interventions CF, WC, CF + WC, or the control group (CG). The subsequent central allocation procedure was managed by the coordinating study center in Hamburg to guarantee allocation concealment. Randomization took place once the standardized baseline assessment and baseline diagnostics were complete.

### Interventions

CF is a face-to-face, psychodynamic, family-centered intervention delivered in eight sessions over six months (T1-T2). It targets mental health issues faced by families with children diagnosed with a rare disease, addressing key topics like disease management, family social networks, family relationships, and professional family support. CF was originally developed, tested, and implemented in a previous pilot project, ‘Chrokodil’ (chronically ill children and their quality of life within the family), and adapted specifically for children with rare diseases and their families [13]. The central target parameters were related to the mental health of all family members and their HRQoL and satisfaction. Nevertheless, given the typically high and varied costs associated with rare diseases in children, health economic analyses were confined to the parents. Modifications for online delivery of face-to-face sessions (e.g. virtual interviews/meetings) were made during the COVID-19 pandemic while retaining essential structures of the intervention. WC is a manualized supportive online intervention designed for parents utilizing cognitive-behavioral writing therapy principles to reduce psychological distress, including symptoms of anxiety, depression, and stress [14]. Parents completed 12 standardized writing tasks over approximately 12–14 weeks, with one 45-minute writing task per week, using a secure internet platform under the supervision of trained therapists. Task content included exposition, cognitive restructuring, and problem-solving. Only one parent per family was permitted to participate in WC, and this parent was selected by the family.

All parents were assessed for psychological stress at baseline as part of a therapist interview. The CG did not receive either study intervention. In the CG, data were collected from all participating parents, thus, families could contribute data from either one or both parents. Routine healthcare services remained available to all study participants.

### Perspective and scope

The primary outcomes of the health economic evaluation were costs and QALYs among parents caring for their children with rare diseases. Incremental costs and incremental QALYs were calculated for each intervention group (CF, WC, CF + WC) compared to the CG. The unit of analysis was the individual parent. Accordingly, all costs and QALYs were measured and analyzed on a per-parent basis across all study groups, irrespective of whether the intervention was delivered to an individual parent (WC) or to the family (CF and CF + WC). Outcomes were not aggregated at the family level. To account for the potential non-independence of observations from parents belonging to the same family, family correlation was explicitly considered in the statistical analysis. Depending on study participation, families contributed data from one or both parents, and all participating parents in the health economic evaluation population were included in the analyses. An intervention was considered dominant if it was both more effective and less costly than the comparator. The analysis was conducted over an 18-month period, considering costs from the SHI payer perspective. This perspective encompasses all healthcare expenditure covered by SHI in Germany, and explicitly includes sickness benefit payments.

### Quality-adjusted life years

In health economics, the QALY is an established unit of measurement for HRQoL which represents one year of life in perfect health. The fundamental question in health economic analysis is the additional cost of gaining a QALY [15]. The concept enables the comparison of various healthcare interventions based on patients’ HRQoL [16]. The HRQoL was measured using the EQ-5D-3L, which is a validated questionnaire that assesses five dimensions: mobility, self-care, usual activities, pain/discomfort, and anxiety/depression. Each dimension is rated as causing ‘no’, ‘some’, or ‘extreme problems’. The questionnaire theoretically generates 3^5^=243 different health states [11, 17]. Utility scores were calculated using the German value set based on the Time-Trade Off Evaluation [18], and QALYs were computed using the area under the curve method [19].

### Costs

Total study costs encompass both intervention costs and total healthcare expenses covered by the participants’ SHI funds during the study period. The costs of SHI include outpatient and inpatient care, institutional outpatient clinics, prescribed drugs, remedies, rehabilitation, and sickness benefit payments. These costs were aggregated for each insured person across all sectors from their baseline measurement to the last measurement date at T4.

The interventions are innovative forms of care that are not routinely reimbursed by the SHI system. Consequently, the intervention costs were based on the reimbursement rates agreed upon with the SHI through selective contracts, reflecting the actual reimbursement amounts from the SHI payer perspective. For example, CF incurred €840 per family, shared proportionally among all family members, equating to €210 per person for a hypothetical family of four. For WC, an amount of €400 per participating parent was agreed upon. These costs were then summed up for families engaged in CF + WC.

### Discount rate

Both costs and QALYs were discounted at a rate of 3%, starting after the initial observation year [20]. Discounted 18-month cost and QALY estimates were annualized (multiplied by 12/18) assuming a constant rate of accrual over the observation period.

### Data preparation

SHI claims data were collected at multiple points within the healthcare system for billing and documentation purposes, rather than for research purposes. The data were therefore extensively validated in accordance with “Good Practice of Secondary Data Analysis” guidelines [21]. To ensure precise cost assessments, costs were calculated daily between the measurement intervals, such as between T1 and T2, reflecting service utilization during these periods. In instances where costs overlapped, they were proportionally allocated to the relevant intervals. A one-year pre-observation period (T0-T1) was employed to ensure the comparability of costs with baseline measurements. Missing HRQoL and cost data were addressed using multiple imputation by chained equations (MICE) based on fully conditional specification and predictive mean matching (PMM). Imputations were performed separately within each treatment group to preserve the randomized group structure. Twenty imputed datasets were generated. Regression models were estimated separately within each imputed dataset and parameter estimates were pooled according to Rubin’s rules. The imputation model included all HRQoL and cost variables from all assessment periods, as well as age and gender. Imputation was performed separately for the ITT-SHI population and sensitivity analysis population.

### Statistical analysis

The primary analysis was conducted in the ITT-SHI population, as defined in the Study Design and Sample section. Missing cost and HRQoL data were handled using MICE, as described above. Continuous variables were described using means, medians, standard deviations (SD), standard errors (SE) for pooled imputed values, 25%-percentiles (Q1) and 75%-percentiles (Q3), and minimum and maximum values.

Descriptive mean costs and QALYs were pooled across the imputed datasets and were unadjusted. For the primary and sensitivity analyses, total discounted QALYs over 18 months were analyzed using linear regression, with treatment group and baseline EQ-5D-3 L utility as explanatory variables and CG as the reference group. Baseline adjustment followed the approach described by Manca et al. (2005) [22]. The intervention coefficients represented baseline-adjusted mean differences in total QALYs relative to CG. Total discounted costs were analyzed using a generalized linear model (GLM) with a Gamma distribution and log link to account for the right-skewed distribution of cost data. For both regression models, family-clustered (CR2) robust standard errors accounted for within-family correlation. Model estimates were pooled across imputed datasets using Rubin’s rules. For each intervention group, incremental costs were calculated as the difference in pooled unadjusted mean total discounted costs, including the intervention costs, relative to CG. Incremental QALYs were obtained from the baseline-adjusted linear regression model. Incremental cost-effectiveness ratios (ICERs) were calculated by dividing incremental costs by baseline-adjusted incremental QALYs. Where appropriate, numerical ICERs were reported as a summary measure of cost-effectiveness. These ICERs represent pairwise comparisons with CG rather than a sequential analysis along the cost-effectiveness frontier.

No adjustment for multiple testing was conducted and no interim analyses were performed. We performed data preparation and analysis using SAS 9.4 for Windows (SAS Institute Inc., Cary, NC, USA) and R version 4.6.0.

As the ICER is a point estimate, it does not account for the stochastic uncertainty of the data [23]. Accordingly, a non-parametric family-cluster bootstrap was conducted for each intervention group compared with CG. Although the individual parent was the unit of analysis, the bootstrap was performed at the family level because families could contribute data from either one or two participating parents. The family identifier was used as the clustering variable, with each family constituting one cluster. Families with only one participating parent were treated as clusters of size one. To account for uncertainty arising from multiple imputation, 500 bootstrap replications were generated within each of the 20 imputed datasets, resulting in a total of 10,000 bootstrap replications for each comparison. In a non-parametric bootstrapping approach, a sample of observations is repeatedly drawn with replacement from the study population [24]. Non-parametric bootstrapping is a standard procedure for conducting cost-effectiveness analyses, as it quantifies the sampling uncertainty of costs and effects [25–27]. To estimate the probability of cost-effectiveness, we used cost-effectiveness acceptability curves (CEACs) and varied the willingness-to-pay (WTP) thresholds from €0 to €50,000 [28–30]. As Germany has no official WTP threshold, €50,000 per QALY was used as a pragmatic reference value. To complement the cost-effectiveness analyses, rank probabilities were calculated using net monetary benefit [31]. For each bootstrap replication, all four study groups were ranked according to their net benefit. The probability of each study group ranking first was plotted across WTP thresholds, and probabilities of achieving each of the four ranks at €50,000 per QALY were presented using rankograms.

### Sensitivity analyses

The primary analysis was repeated in a restricted population. Only participants from the ITT-SHI population who had at least two observed EQ-5D-3 L assessments were included in this sensitivity analysis. This analysis assessed the sensitivity of the findings to the inclusion of participants with limited observed HRQoL data and the greater reliance on imputation in the ITT-SHI population. Missing data were imputed separately in the restricted population using the same imputation approach. The same statistical models, family-clustered CR2 robust standard errors, ICER definition, and non-parametric family-cluster bootstrap procedures were applied as in the primary analysis.

Baseline characteristics were compared between participants included in the sensitivity analysis and the additional participants included in the ITT-SHI population. Additional leave-one-out analyses assessed the influence of individual extreme cost observations on the cost-effectiveness results. All observations were retained in the primary and the sensitivity analysis. The identified observations were excluded only in the supplementary leave-one-out analyses.

## Results

**Fig. 1:**
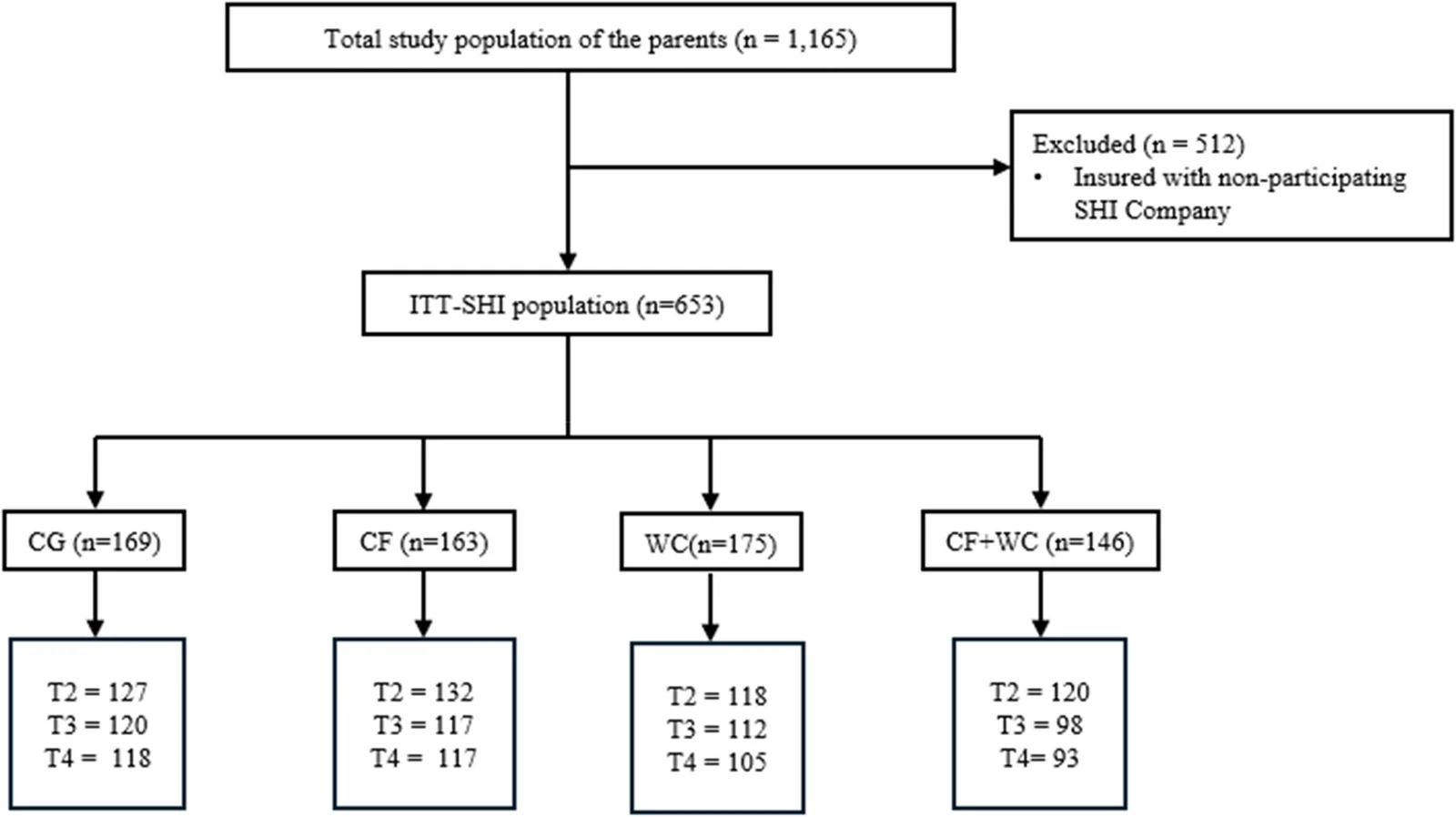
Study Population. CF: Intervention CARE-FAM, CF + WC: Intervention CARE-FAM + WEP-CARE, CG: Control group, EQ-5D-3 L: EuroQol (questionnaire)-5 Dimensions-3 Level, WC: Intervention WEP-CARE, SHI: Statutory health insurance

The initial study population consisted of 1165 parents. After excluding 512 individuals insured with non-participating SHI funds, the ITT-SHI population included 653 parents and formed the primary analysis population. Of these, 106 individuals had fewer than two observed EQ-5D-3 L assessments. The remaining 547 parents formed the population for the sensitivity analysis (Fig. 1).

Multiple imputation was used to impute missing EQ-5D-3 L scores for 156 individuals at T2 (23.9%), 206 individuals at T3 (31.5%), and 220 individuals at T4 (33.7%). Cost data required imputation for 91 individuals during T2-T3 (13.9%) and for 242 individuals during T3-T4 (37.1%). Additionally, age data were imputed for 26 individuals (4.0%). The mean participant age was 39.4 years (SD 7.2 years), and 396 (60.6%) were female. Of the participants, 364 (55.7%) had a high level of education, and 536 (82.1%) were married or living with a partner. Regarding family structure, 53.0% had two children and 27.4% had one child. At baseline, 64 individuals (9.8%) reported a mental illness. The mean BSI Global Severity Index was 0.3773 (SD 0.3775), the mean BSI Anxiety subscale score was 0.3243 (SD 0.3983) and the mean EQ-5D-3 L score at baseline (T1) was 0.9198 (SD 0.1433). During the pre-observation period, participants incurred mean costs of €4090 (SD €15086) (Table 1). There were no relevant differences between the intervention groups and the CG regarding baseline characteristics. Comparison of baseline characteristics between participants included in the sensitivity analysis (*n* = 547) and the additional participants included in the ITT-SHI population (*n* = 106) showed no statistically significant differences in age, baseline HRQoL, BSI Global Severity Index, BSI Anxiety subscale score, education level, relationship status or the presence of mental illness at baseline. However, the additional participants included in the ITT-SHI population were more likely to be male (50.0% vs. 37.3%, *p* = 0.014) and to have three or more children (28.6% vs. 17.6%, *p* = 0.002) (Supplementary Table 2).

**Table 1:** Baseline Characteristics

	CG (*n* = 169)	CF (*n* = 163)	WC (*n* = 175)	CF + WC (*n* = 146)	Total (*n* = 653)
Gender					
Male [n (%)]	65 (38.5)	68 (41.7)	71 (40.6)	53 (36.3)	257 (39.4)
Female [n (%)]	104 (61.5)	95 (58.3)	104 (59.4)	93 (63.7)	396 (60.6)
Age					
Mean (SD)	39.2 (7.2)	38.8 (7.8)	40.5 (6.9)	39.0 (6.9)	39.4 (7.2)
Median (Q1; Q3)	38.5 (34.0; 44.0)	37.0 (34.0; 42.0)	40.0 (35.0; 45.0)	38.0 (35.0; 44.0)	38.0 (34.0; 44.0)
Min. - Max.	24–60	24–66	23–59	17–55	17–66
Education					
Lowᵃ [n (%)]	13 (7.7)	11 (6.7)	13 (7.4)	16 (11.0)	53 (8.1)
Moderateᵃ [n (%)]	50 (29.6)	48 (29.4)	50 (28.6)	46 (31.5)	194 (29.7)
Highᵃ [n (%)]	94 (55.6)	93 (57.1)	98 (56.0)	79 (54.1)	364 (55.7)
Missing [n (%)]	12 (7.1)	11 (6.7)	14 (8.0)	5 (3.4)	42 (6.4)
Married or with a partner [n (%)]	144 (85.2)	127 (77.9)	146 (83.4)	119 (81.5)	536 (82.1)
Missing [n (%)]	0 (0.0)	1 (0.6)	1 (0.6)	1 (0.7)	3 (0.5)
Number of children [n (%)]					
1	54 (32.0)	53 (32.5)	35 (20.0)	37 (25.3)	179 (27.4)
2	83 (49.1)	81 (49.7)	96 (54.9)	86 (58.9)	346 (53.0)
3	25 (14.8)	19 (11.7)	32 (18.3)	11 (7.5)	87 (13.3)
> 3	7 (4.1)	10 (6.1)	10 (5.7)	12 (8.2)	39 (6.0)
Missing [n (%)]	0 (0.0)	0 (0.0)	2 (1.1)	0 (0.0)	2 (0.3)
Suffering from mental illness [n (%)]	17 (10.1)	12 (7.4)	17 (9.7)	18 (12.3)	64 (9.8)
Missing [n (%)]	1 (0.6)	2 (1.2)	4 (2.3)	6 (4.1)	13 (2.0)
BSI Global Severity Index					
Mean (SD)	0.3932 (0.3675)	0.3967 (0.4024)	0.3828 (0.4182)	0.3297 (0.2999)	0.3773 (0.3775)
Median (Q1; Q3)	0.3019 (0.1321; 0.5283)	0.2453 (0.0943; 0.6038)	0.2642 (0.1132; 0.5094)	0.2264 (0.0943; 0.4906)	0.2642 (0.1132; 0.5283)
Min. - Max.	0.0000–1.9057	0.0000–1.8491	0.0000–2.3208	0.0000–1.3962	0.0000–2.3208
BSI Anxiety subscale score					
Mean (SD)	0.3234 (0.4023)	0.3230 (0.4267)	0.3295 (0.3921)	0.3207 (0.3712)	0.3243 (0.3983)
Median (Q1; Q3)	0.1667 (0.0000; 0.5000)	0.1667 (0.0000; 0.5000)	0.1667 (0.0000; 0.5000)	0.1667 (0.0000; 0.5000)	0.1667 (0.0000; 0.5000)
Min. - Max.	0.0000–3.0000	0.0000–2.5000	0.0000–2.1667	0.0000–1.6667	0.0000–3.0000
EQ-5D-3 L Score (T1)					
Mean (SD)	0.9144 (0.1492)	0.9212 (0.1569)	0.9164 (0.1489)	0.9286 (0.1113)	0.9198 (0.1433)
Median (Q1; Q3)	0.9990 (0.8870; 1.0000)	0.9990 (0.8870; 1.0000)	0.9990 (0.8870; 1.0000)	0.9990 (0.8870; 1.0000)	0.9990 (0.8870; 1.0000)
Min. - Max.	0.1750–1.0000	0.1750–1.0000	0.1970–1.0000	0.2960–1.0000	0.1750–1.0000
Total healthcare costs (T0-T1)					
Mean (SD)	€3725 (€5753)	€3350 (€5090)	€3915 (€12404)	€5470 (€27207)	€4090 (€15086)
Median (Q1; Q3)	€1401 (€401; €4992)	€1346 (€380; €4659)	€1155 (€316; €3864)	€1330 (€574; €3878)	€1304 (€401; €4203)
Min. - Max.	€0 - €31,542	€0 - €33,630	€0 - €149,076	€0 - €323,563	€0 - €323,563

Values are based on observed baseline data. *CF *Intervention CARE-FAM, *CF + WC *Intervention CARE-FAM + WEP-CARE, *CG* Control group, *EQ-5D-3 L* EuroQol (questionnaire)-5 Dimensions-3 Level, *Max* Maximum, *Min* Minimum, *Q1* 25%-percentile, *Q3* 75%-percentile, *SD* Standard deviation, *T0-T1* One-year pre-observation period until baseline, *WC* Intervention WEP-CARE, ^a^Low refers to 9 years of schooling, moderate to 10 years, and high to 12–13 years

Table 2 presents descriptive statistics for the observed, non-imputed EQ-5D-3 L scores and healthcare costs by study group (see Supplementary Table 3 for dimension-specific EQ-5D-3 L values). Mean observed EQ-5D-3 L scores were generally high across all study groups from T2 to T4, ranging from 0.9146 to 0.9433. The number of participants with available observations varied across measurement times and cost periods and is reported separately for each study group in Table 2. Over the 18-month follow-up, mean total costs per parent, excluding intervention costs, were €5311 in the CG, €4385 in the CF group, €5430 in the WC group and €7056 in the CF + WC group. Furthermore, the highest individual total costs were observed in CF + WC (€390,698), followed by WC (€325,725) and CF (€148,861). These costs were primarily attributable to high medication costs incurred by individual participants (€368,052, €307,625 and €108,832, respectively).

Mean costs by healthcare sector for the ITT-SHI population and the sensitivity analysis population are presented in Supplementary Tables 4 and 5. In both populations, outpatient care and medication costs accounted for the largest shares of the overall healthcare costs, whereas rehabilitation and outpatient long-term nursing care contributed comparatively little.

**Table 2:** Observed EQ-5D-3 L scores and total costs per parent by study group

Outcome	Statistic	CG	CF	WC	CF + WC
EQ-5D-3 L score (T2) (6 months)		(*n* = 127)	(*n* = 132)	(*n* = 118)	(*n* = 120)
	Mean (SD)	0.9153 (0.1327)	0.9195 (0.1421)	0.9319 (0.1167)	0.9403 (0.0887)
	Median (Q1; Q3)	0.8870 (0.8870; 1.0000)	0.9990 (0.8870; 1.0000)	0.9990 (0.8870; 1.0000)	0.9990 (0.8870; 1.0000)
	Min.-Max.	0.1970–1.0000	0.1750–1.0000	0.2960–1.0000	0.4990–1.0000
EQ-5D-3 L score (T3) (12 months)		(*n* = 120)	(*n* = 117)	(*n* = 112)	(*n* = 98)
	Mean (SD)	0.9257 (0.1149)	0.9217 (0.1454)	0.9433 (0.1055)	0.9354 (0.1126)
	Median (Q1; Q3)	0.9990 (0.8870; 1.0000)	1.0000 (0.8870; 1.0000)	0.9995 (0.8870; 1.0000)	0.9990 (0.8870; 1.0000)
	Min.-Max.	0.1750–1.0000	0.1750–1.0000	0.2620–1.0000	0.1100–1.0000
EQ-5D-3 L score (T4) (18 months)		(*n* = 118)	(*n* = 117)	(*n* = 105)	(*n* = 93)
	Mean (SD)	0.9146 (0.1267)	0.9261 (0.1529)	0.9329 (0.1286)	0.9272 (0.1445)
	Median (Q1; Q3)	0.9990 (0.8870; 1.0000)	1.0000 (0.8870; 1.0000)	1.0000 (0.8870; 1.0000)	1.0000 (0.8870; 1.0000)
	Min.-Max.	0.2620–1.0000	0.1100–1.0000	0.1750–1.0000	0.1100–1.0000
Total costs per parent (T1-T4) (baseline − 18 months)		(*n* = 169)	(*n* = 163)	(*n* = 175)	(*n* = 146)
	Mean (SD)	€5311 (€10211)	€4385 (€13767)	€5430 (€24924)	€7056 (€33150)
	Median (Q1; Q3)	€1463 (€342; €5371)	€1113 (€354; €3532)	€1649 (€500; €4867)	€1362 (€477; €4764)
	Min.-Max.	€0 - €74,374	€0 - €148,861	€0 - €325,725	€0 - €390,698
Total costs per parent including intervention costs (T1-T4) (baseline − 18 months)		(*n* = 169)	(*n* = 163)	(*n* = 175)	(*n* = 146)
	Mean (SD)	€5311 (€10211)	€4620 (€13767)	€5570 (€24916)	€7433 (€33150)
	Median (Q1; Q3)	€1463 (€342; €5371)	€1347 (€589; €3766)	€1862 (€647; €4956)	€1704 (€887; €5006)
	Min.-Max.	€0 - €74,374	€234 - €149,095	€0 - €325,725	€242 - €391,074
Total costs per parent (T1-T2) (baseline − 6 months)		(*n* = 161)	(*n* = 155)	(*n* = 174)	(*n* = 144)
	Mean (SD)	€2603 (€5008)	€2628 (€8520)	€2388 (€9789)	€3765 (€18812)
	Median (Q1; Q3)	€672 (€218; €2530)	€537 (€133; €1705)	€743 (€168; €2073)	€572 (€153; €1884)
	Min.-Max.	€0 - €32,594	€0 - €78,218	€0 - €124,859	€0 - €218,923
Total costs per parent (T2-T3) (6 months − 12 months)		(*n* = 144)	(*n* = 136)	(*n* = 156)	(*n* = 126)
	Mean (SD)	€1835 (€4632)	€1443 (€4460)	€2054 (€9026)	€2694 (€13905)
	Median (Q1; Q3)	€384 (€79; €1513)	€256 (€64; €803)	€482 (€46; €1573)	€354 (€67; €1434)
	Min.-Max.	€0 - €33,616	€0 - €39,318	€0 - €108,704	€0 - €152,947
Total costs per parent (T3-T4) (12 months − 18 months)		(*n* = 106)	(*n* = 97)	(*n* = 116)	(*n* = 92)
	Mean (SD)	€2020 (€4414)	€1145 (€3602)	€1849 (€8712)	€1615 (€4385)
	Median (Q1; Q3)	€390 (€63; €1450)	€179 (€43; €647)	€522 (€40; €1120)	€305 (€42; €1364)
	Min.-Max.	€0 - €31,519	€0 - €31,325	€0 - €92,163	€0 - €29,336

*CF* Intervention CARE-FAM, *CF + WC* Intervention CARE-FAM + WEP-CARE, *CG* Control group, *EQ-5D-3 L* EuroQol (questionnaire)-5 Dimensions-3 Level, *Max* Maximum, *Min* Minimum, *Q1* 25%-percentile, *Q3* 75%-percentile, *SD* Standard deviation, *WC* Intervention WEP-CARE. All values are based on observed data before imputation. n indicates the number of participants with an available observed value at the respective EQ-5D-3 L measurement time or cost period. Costs are presented as undiscounted total costs without intervention costs (for T1-T4, total costs are additionally presented including intervention costs). Total costs for T1-T4 represent the sum of available observed period costs

Mean total discounted costs and QALYs per parent over the 18-month observation period (T1 to T4) are presented in Table 3. Mean total discounted costs per parent were €6713 (SE €946) for CG, €5309 (SE €1133) for CF, €5955 (SE €1874) for WC and €8277 (SE €2774) for CF + WC. The GLM indicated lower estimated mean costs for CF and WC compared with CG, with cost ratios of 0.7910 and 0.8877, whereas CF + WC had higher estimated mean costs, with a cost ratio of 1.2330. However, none of these differences were statistically significant. Unadjusted mean total discounted QALYs per parent were 1.3716 (SE 0.0119) for CG, 1.3823 (SE 0.0145) for CF, 1.3930 (SE 0.0111) for WC and 1.3970 (SE 0.0112) for CF + WC (Table 3). After adjustment for baseline EQ-5D utility, the estimated mean differences in total QALYs compared with CG were 0.0058 for CF, 0.0199 for WC and 0.0143 for CF + WC. None of these differences were statistically significant. A comparison of imputed and observed values of EQ-5D-3 L scores and total costs is presented in Supplementary Tables 6 and 7.

**Table 3:** Results of the cost-effectiveness analysis in the ITT-SHI population (*n* = 653)

	CG (*n* = 169)	CF (*n* = 163)	WC (*n* = 175)	CF + WC (*n* = 146)
Mean total Costs (SE)^a^	€6713 (€946)	€5309 (€1133)	€5955 (€1874)	€8277 (€2774)
GLM Cost ratio (95% CI)^b^ ; *p*-value	Reference	0.7910 (0.4759–1.3145); *p* = 0.3655	0.8877 (0.4520–1.7433); *p* = 0.7294	1.2330 (0.5993–2.5368); *p* = 0.5693
∆ Mean total Costs	Reference	-€1404	-€758	€1564
Mean annual Costs (SE)^d^	€4476 (€631)	€3539 (€755)	€3970 (€1249)	€5518 (€1850)
∆Mean annual Costs ^d^	Reference	-€937	-€506	€1042
Mean total QALYs (SE)^a^	1.3716 (0.0119)	1.3823 (0.0145)	1.3930 (0.0111)	1.3970 (0.0112)
Adjusted mean difference in total QALYs (95% CI); *p*-value ^*c*^	Reference	0.0058 (-0.0189- 0.0305); *p* = 0.6453	0.0199 (-0.0013- 0.0412); *p* = 0.0655	0.0143 (-0.0114-0.0400); *p* = 0.2744
∆ Mean total QALYs	Reference	0.0107	0.0214	0.0254
Mean annual QALYs (SE)^d^	0.9144 (0.0079)	0.9215 (0.0097)	0.9287 (0.0074)	0.9313 (0.0075)
∆Mean annual QALYs ^d^	Reference	0.0071	0.0143	0.0169
ICER vs. CG (∆Cost/∆QALY)^e^	Reference	Dominates CG	Dominates CG	€109,371/QALY

*CF* Intervention CARE-FAM, *CF + WC* Intervention CARE-FAM + WEP-CARE, *CG* Control group, *QALY* Quality-Adjusted Life Year, *SE* Standard error, *WC* Intervention WEP-CARE, ^a^Unadjusted mean total discounted costs including intervention costs and unadjusted mean discounted QALYs per parent during the 18-month observation period (T1 to T4), ^b^Cost ratios derived from a GLM (log link) and family-cluster-robust standard errors, pooled across m = 20 MI-datasets using Rubin’s Rules. ^c^Baseline-adjusted mean differences in total discounted QALYs relative to CG were estimated using linear regression with treatment group and baseline EQ-5D-3 L utility as explanatory variables and family-clustered CR2 robust standard errors. Estimates were pooled across 20 MI-datasets using Rubin’s Rules. ^d^Mean unadjusted annual discounted costs or QALYs per parent (T1 to T4); annualized values assume uniform costs over the 18-month observation period. ^e^ICERs were calculated by dividing the difference in pooled unadjusted mean total discounted costs by the baseline-adjusted mean difference in total discounted QALYs

The unadjusted mean annual discounted QALYs per parent were 0.9144 (SE 0.0079) for CG. Compared with the CG, the unadjusted incremental mean annual discounted QALYs per parent were 0.0071 in CF, 0.0143 in WC, and 0.0169 in CF + WC. Mean annual discounted costs per parent were highest for CF + WC at €5518 (SE €1850) and lowest for CF at €3539 (SE €755), compared with €4476 (SE €631) for CG. Relative to CG, incremental mean annual discounted costs were lower by €937 for CF and €506 for WC, but higher by €1042 for CF + WC.

**Fig. 2:**
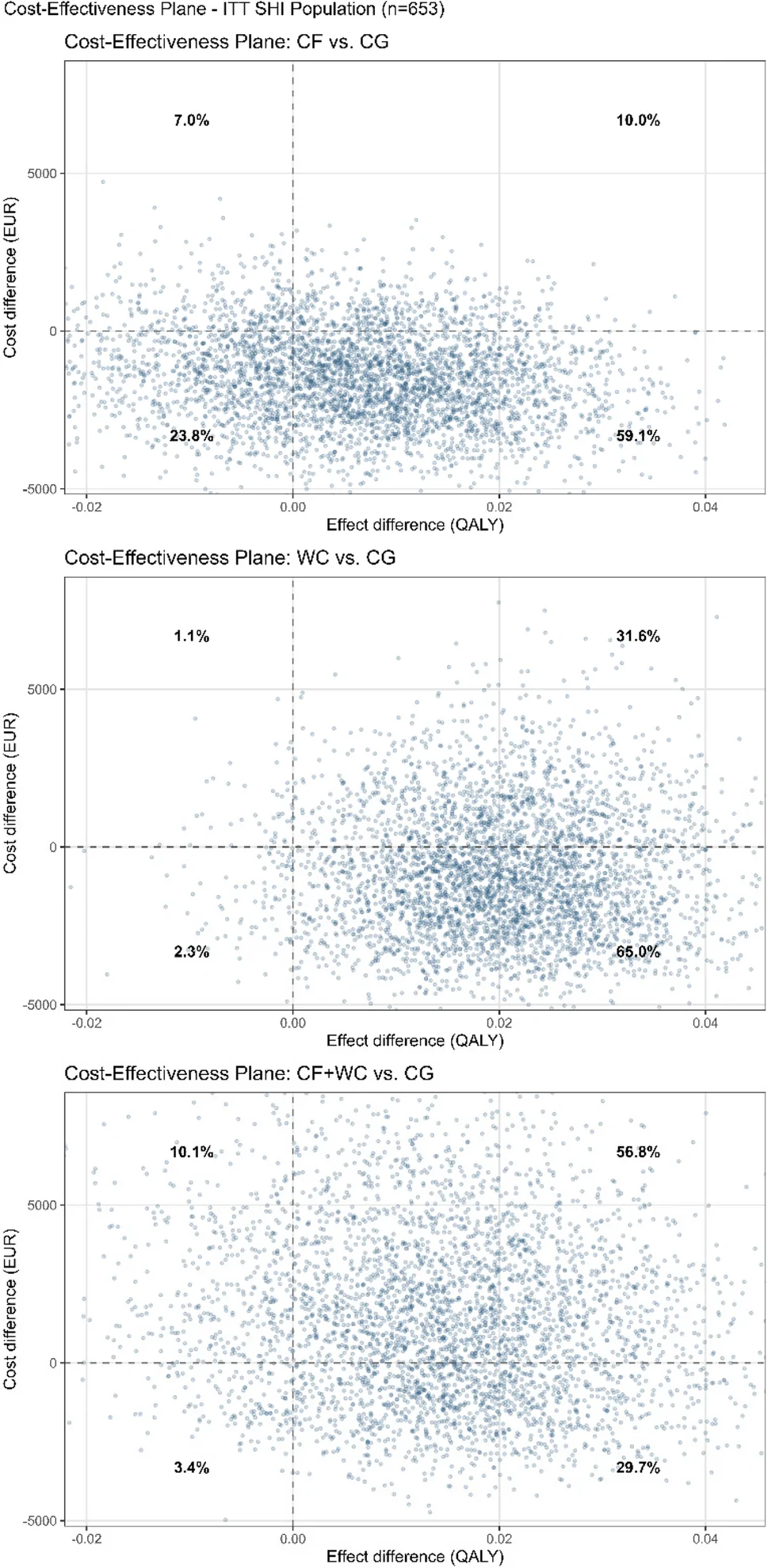
Cost-effectiveness planes of interventions CF, WC, and CF + WC compared with CG. CF: Intervention CARE-FAM, CF + WC: Intervention CARE-FAM + WEP-CARE, CG: Control group, QALY: Quality-Adjusted Life Year, WC: Intervention WEP-CARE

Based on the point estimates of incremental costs and baseline-adjusted incremental QALYs, CF and WC dominated CG, as both were associated with lower mean discounted costs and positive baseline-adjusted QALY differences. CF + WC was associated with higher mean discounted costs and a positive baseline-adjusted QALY difference, resulting in an ICER of €109,371 per QALY gained. Figure 2 visualizes the 10,000 ICER estimations using bootstrapping for CF, WC, and CF + WC compared with CG, showing the distribution of bootstrap replications across the four cost-effectiveness quadrants. For CF compared with CG, 59.1% of the 10,000 bootstrap replications fell within the south-east quadrant, indicating a 59.1% probability that CF was dominant (i.e. more effective and less costly) than CG. The corresponding proportions for WC and CF + WC were 65.0% and 29.7%, respectively. For CF + WC, most replications (56.8%) fell within the north-east quadrant, indicating higher QALYs but also higher costs than CG.

**Fig. 3:**
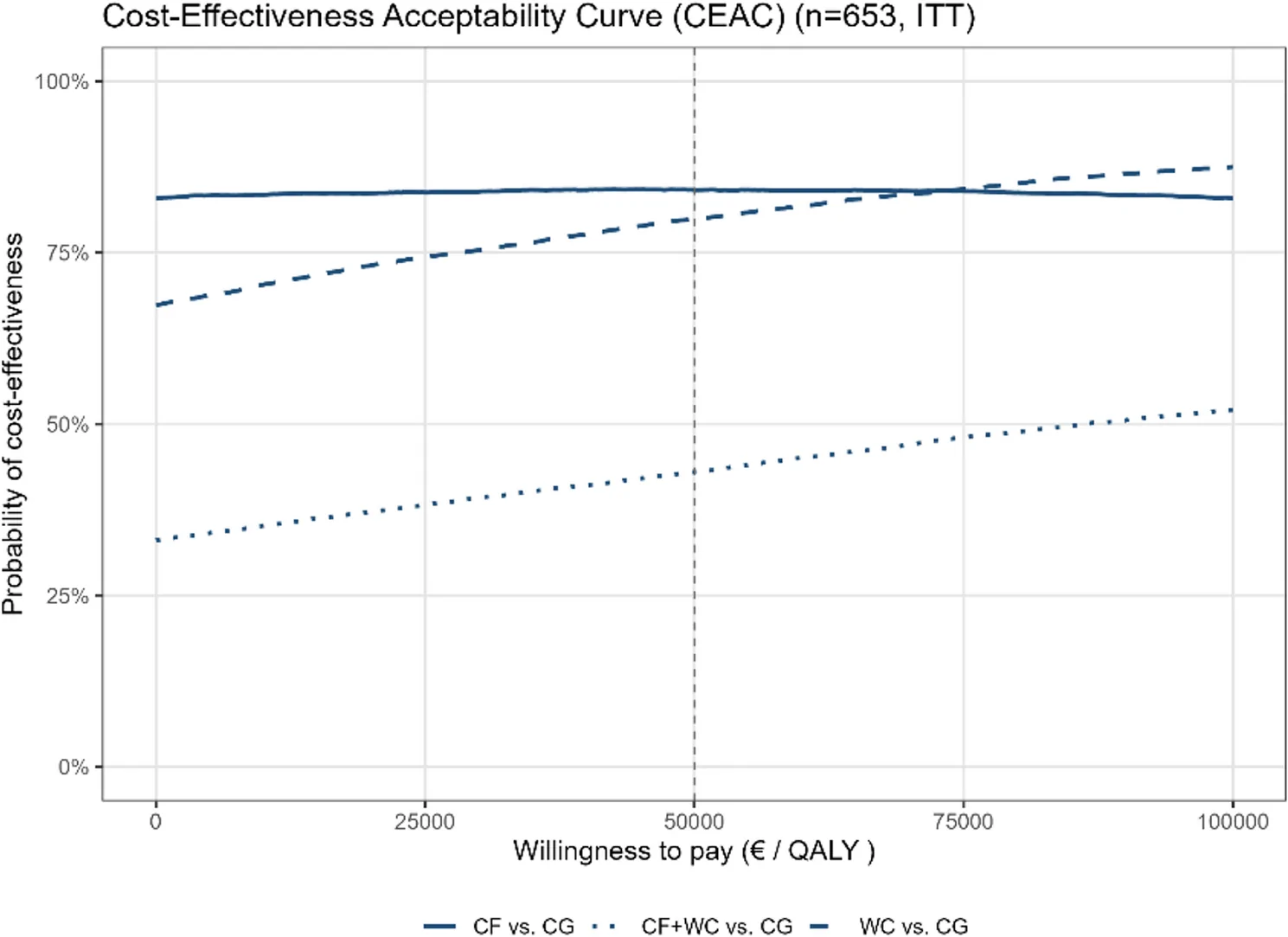
Cost-effectiveness acceptability curves for interventions CF, WC and CF + WC compared to the CG. CF: Intervention CARE-FAM, CF + WC: Intervention CARE-FAM + WEP-CARE, CG: Control group, WC: Intervention WEP-CARE

Figure 3 shows the probabilities of cost-effectiveness using CEACs. These curves display the probability of cost-effectiveness for all interventions compared with CG at different WTP thresholds per QALY gained. As the WTP increased, the probability of cost-effectiveness increased for WC and CF + WC, whereas it remained relatively stable for CF. At a WTP of €50,000 per QALY, the probability of being cost-effective was 84.2% for CF, 79.9% for WC and 43.0% for CF + WC.

**Fig. 4:**
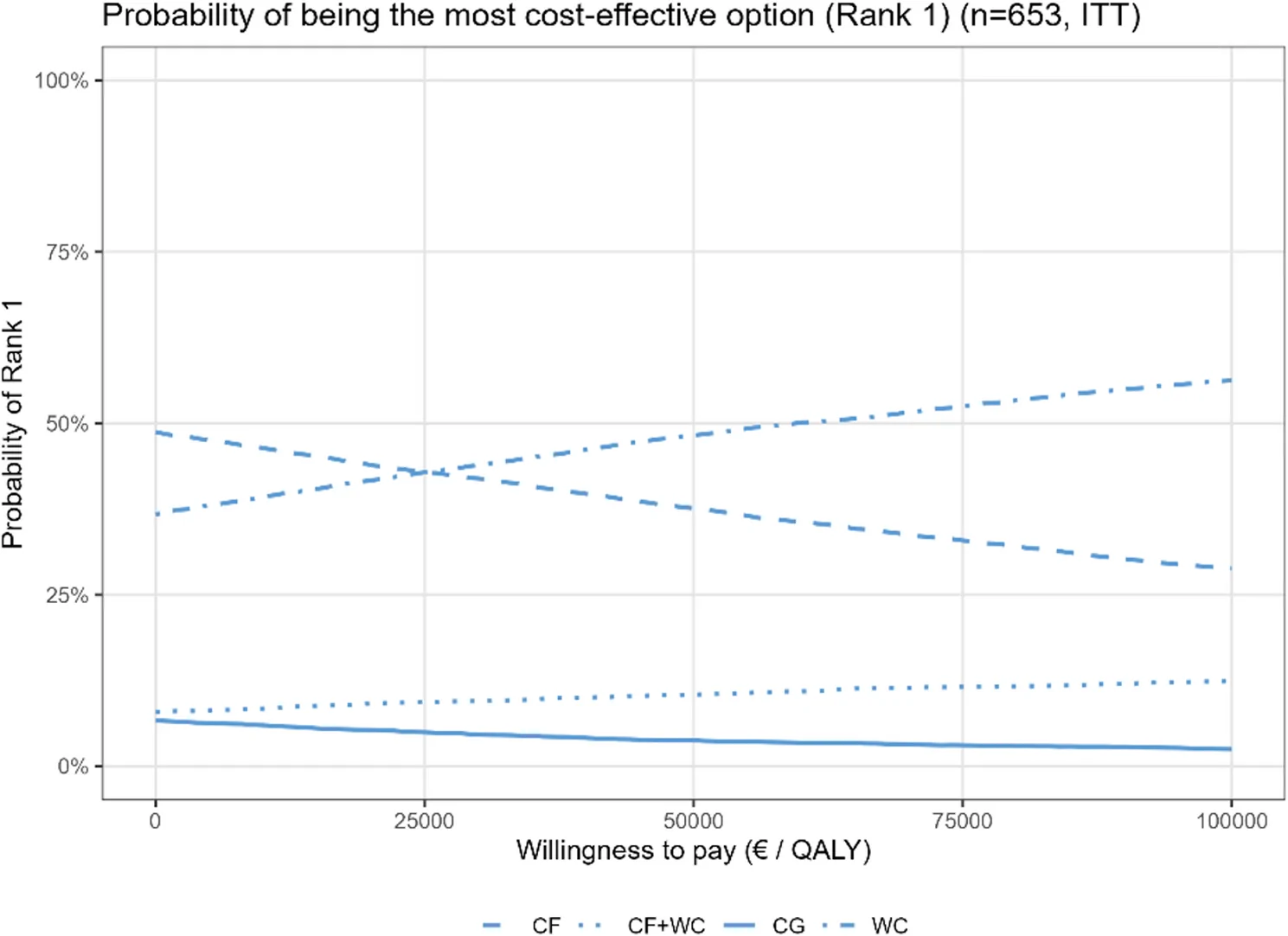
Probability of being the most cost-effective option across willingness-to-pay-thresholds (WTP). CF: Intervention CARE-FAM, CF + WC: Intervention CARE-FAM + WEP-CARE, CG: Control group, WC: Intervention WEP-CARE

**Fig. 5:**
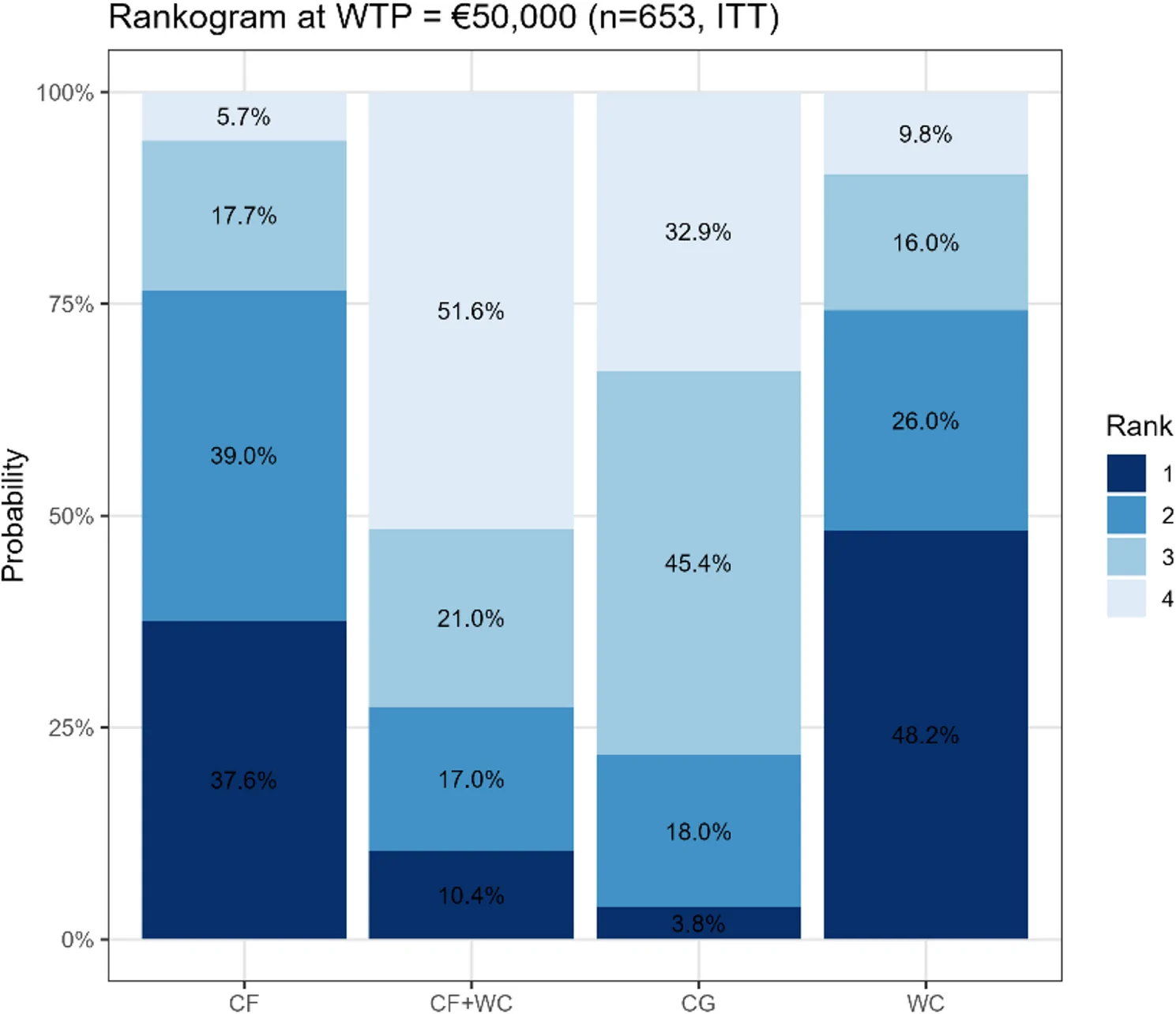
Rankogram at a WTP of €50,000 per QALY. CF: Intervention CARE-FAM, CF + WC: Intervention CARE-FAM + WEP-CARE, CG: Control group, WC: Intervention WEP-CARE

At a WTP threshold of €50,000 per QALY, the rank probabilities indicated that the WC intervention had the highest probability of being the most cost-effective strategy (48.2%), followed by CF (37.6%) and CF + WC (10.4%), whereas the CG had a 3.8% probability of ranking first (Fig. 4). The rankogram further showed that CF had the lowest probability of being ranked last (5.7%), while CF + WC had the highest probability of being ranked fourth (51.6%), followed by CG (32.9%) and WC (9.8%) (Fig. 5).

### Sensitivity analyses

The sensitivity analysis included 547 participants and yielded results comparable to the primary analysis for CF and WC, whereas results differed for CF + WC. Mean total discounted costs per parent were €6804 (SE €1094) for CG, €5639 (SE €1292) for CF, €6372 (SE €2419) for WC and €6098 (SE €1003) for CF + WC. Cost ratios relative to CG were 0.8300 for CF, 0.9382 for WC and 0.8964 for CF + WC. None of these differences were statistically significant.

Unadjusted mean total discounted QALYs per parent were 1.3729 (SE 0.0123), 1.3787 (SE 0.0163), 1.3916 (SE 0.0132) and 1.3964 (SE 0.0115) for CG, CF, WC and CF + WC, respectively. After adjustment for baseline EQ-5D-3 L utility, the estimated mean differences in total QALYs compared with CG were 0.0029 for CF, 0.0190 for WC and 0.0163 for CF + WC. None of these differences were statistically significant. Based on the unadjusted incremental mean costs and baseline-adjusted QALY differences, all three interventions dominated CG in the sensitivity analysis. Results of the sensitivity analysis are presented in Supplementary Table 8.

In the sensitivity analysis population, the distribution of bootstrap replications showed broadly similar patterns to the primary analysis for CF and WC. For CF, 48.1% of bootstrap replications were located in the south-east quadrant, compared with 57.2% for WC. In contrast to the primary analysis, the distribution for CF + WC shifted towards the south-east quadrant (59.6%), indicating higher QALYs and lower costs compared with CG (Supplementary Fig. 1). At a WTP of €50,000 per QALY, the probability of being cost-effective was 76.1% for CF, 72.2% for WC and 82.0% for CF + WC (Supplementary Fig. 2). Compared with the primary analysis, the probabilities of being cost-effective decreased for CF and WC, whereas the probability for CF + WC increased. At a WTP of €50,000 per QALY, WC had the highest probability of being ranked first (42.6%), followed by CF and CF + WC (both 26.6%), while CG ranked first in 4.1% of bootstrap replications (Supplementary Fig. 3). CF + WC had a higher probability of ranking first than in the primary ITT-SHI analysis (26.6% vs. 10.4%). The rankogram showed that CF + WC had the lowest probability of being ranked last (8.9%), compared with 51.6% in the primary analysis. In the sensitivity analysis, CG had the highest probability of being ranked last (51.4%) (Supplementary Fig. 4). As the probability of cost-effectiveness for CF + WC differed substantially between the primary ITT-SHI analysis and the sensitivity analysis, a leave-one-out analysis was performed to assess the influence of the previously described participant with exceptionally high healthcare costs in the CF + WC group. Excluding this participant increased the probability that CF + WC was cost-effective at a WTP of €50,000 per QALY from 43.0% to 90.9%. The distribution of bootstrap replications shifted towards the south-east quadrant, which contained 71.3% of bootstrap replications after exclusion. These findings suggest that the observed difference between the primary and sensitivity analyses was largely driven by a single extreme cost observation. A similar influence of an extreme cost observation was evident for WC in the sensitivity analysis population: excluding one participant with total healthcare costs of €325,725 increased the probability of cost-effectiveness from 72.2% to 99.9% at a WTP of €50,000 per QALY.

## Discussion

### Main findings

The aim of this study was to evaluate the cost-effectiveness of two innovative interventions, CF and WC, and their combination, for parents caring for children with rare diseases. Over the 18-month follow-up period, CF and WC in the primary ITT-SHI analysis were associated with lower mean costs and positive baseline-adjusted QALY differences relative to CG, resulting in dominance. In contrast, CF + WC was associated with higher mean costs and a positive baseline-adjusted QALY difference, resulting in an ICER of €109,371 per QALY gained. WC had the largest baseline-adjusted QALY gain, although none of the differences in costs or baseline-adjusted QALYs were statistically significant. However, the ICER point estimates alone do not capture statistical uncertainty, as reflected in the CEACs. At a WTP of €50,000 per QALY, the probability of cost-effectiveness compared with CG was estimated at 84.2% for CF, 79.9% for WC and 43.0% for CF + WC. CEACs offer a more intuitive tool for decision-makers compared to confidence intervals, simplifying data interpretation [32]. Because three interventions were evaluated simultaneously, we additionally estimated rank probabilities. In the primary analysis, WC had the highest probability of being the most cost-effective strategy (48.2%), followed by CF (37.6%) and CF + WC (10.4%), whereas CG had a 3.8% probability of ranking first.

The sensitivity analysis showed similar findings for CF and WC, which remained dominant compared with CG. In contrast to the primary analysis, CF + WC was also associated with lower mean costs and a positive baseline-adjusted QALY difference, resulting in dominance over CG. Accordingly, its probability of ranking first increased from 10.4% to 26.6%, whereas the corresponding probabilities decreased for CF and WC. Nevertheless, the leave-one-out analysis showed that excluding a single participant with high healthcare costs from the ITT-SHI population increased the probability of cost-effectiveness for CF + WC from 43.0% to 90.9% at a WTP of €50,000 per QALY. These findings indicate that the cost-effectiveness results for CF + WC were sensitive to an individual extreme cost observation. This observation may also help explain the bimodal distribution observed in its cost-effectiveness plane.

Previous studies have evaluated WC’s impact, though they did not focus on QALY gains. For instance, Fidika et al. (2015) reported increased HRQoL for caregivers of children with cystic fibrosis [14], while Tutus et al. (2024) found significant reductions in anxiety and depression among parents who received WC while caring for chronically ill children. However, differences in HRQoL were not significant [33].

As outlined in the Introduction, parents of children with rare diseases consistently have lower HRQoL compared with parents of healthy children or population norms [5–8]. In contrast, participants in our study already showed relatively high baseline EQ-5D-3 L utility values. This discrepancy may reflect differences in the HRQoL instruments used. The relatively high baseline HRQoL may also explain the modest gains observed in our study. Evidence from trial-based cost-utility analyses of psychosocial interventions targeting parents remains limited. However, our findings are broadly consistent with previous economic evaluations of psychosocial interventions for parents. For example, Shields et al. (2025) evaluated a psychosocial intervention for parents of children with autism in a trial-based cost-effectiveness analysis and similarly reported modest QALY gains despite improvements in caregiver outcomes, accompanied by considerable uncertainty regarding cost-effectiveness [34]. While the UK’s National Health Service considers a threshold of £20,000-£30,000 per QALY to be cost-effective, Germany does not adhere to a fixed amount per QALY, though most Western countries generally consider higher thresholds acceptable [35, 36]. Given its emphasis on physical health, instruments other than the EQ-5D may be better suited to capturing the HRQoL of the study population. However, in Germany it remains the only questionnaire that allows for the calculation of QALYs.

Recent literature has increasingly emphasized the importance of incorporating caregiver HRQoL into pediatric economic evaluations, because childhood illness may substantially affect the health and well-being of family caregivers [37]. While caregiver spillover effects have traditionally been evaluated in studies of child-focused interventions [38, 39], our study provides complementary evidence by evaluating mental health support interventions delivered directly to caregivers. By incorporating parental HRQoL and QALYs into a cost-utility analysis, our findings contribute to the growing evidence on the broader health and economic consequences of caring for a child with a rare disease.

The study results may have been influenced by the COVID-19 pandemic. Studies have shown that parents caring for children with rare diseases experienced increased psychosocial burden during the pandemic, which may have negatively affected their HRQoL independent of the intervention [40, 41]. Therefore, potential improvements in HRQoL may have been attenuated. Furthermore, the pandemic may have affected healthcare utilization and consequently healthcare costs. As these effects were not explicitly measured or adjusted for, their impact on the study findings cannot be quantified.

The generalizability of our findings should be interpreted with caution. The economic evaluation was conducted within the German statutory health insurance system using German claims data. This may limit the direct transferability of the cost estimates to other healthcare systems. However, the psychosocial challenges experienced by parents caring for children with rare diseases are similar across many countries, suggesting that the observed effects on HRQoL may be applicable beyond the German setting. Furthermore, as all participants were insured within the German SHI funds, the findings are representative of the German SHI population.

### Strengths and limitations

This study contributes to the evidence on randomized controlled trials investigating the HRQoL of family caregivers of children with rare diseases receiving face-to-face interventions such as CF. The study’s randomized controlled trial design ensures comparability between groups without relevant baseline differences. The use of SHI data is a methodological strength, providing a reliable measure of cost data through actual service utilization without reliance on self-reported data, thus minimizing recall bias and underreporting [42–44].

A further methodological strength was the inclusion of all participating parents, allowing families to contribute data from either one or both parents. This retained available information, while within-family correlation was accounted for using family-level clustering methods in the statistical analyses. Nonetheless, several limitations must be acknowledged. Typically, a power calculation is performed for the primary efficacy endpoint of a clinical trial. Since this health economic evaluation was a secondary analysis, the sample size may be insufficient to detect a statistically significant effect [45]. With mean baseline EQ-5D-3 L values ranging from 0.9144 (SD 0.1492) to 0.9286 (SD 0.1113), HRQoL was relatively high, potentially indicating a ceiling effect [46]. Furthermore, the use of the EQ-5D-3 L rather than the EQ-5D-5 L may have contributed to the observed ceiling effects. Studies have shown that the EQ-5D-5 L demonstrates improved sensitivity and reduced ceiling effects compared with the EQ-5D-3L [47, 48]. While the EQ-5D-3 L was the instrument specified in the original study protocol, future studies should consider using the EQ-5D-5 L, which may be more sensitive to changes in HRQoL. The study population, primarily composed of younger parents (median age 38), was not chosen based on particular indications, possibly accounting for the elevated baseline HRQoL values.

The primary ITT-SHI analysis included all 653 participants in the economic evaluation population. Of these, 106 participants were excluded from the restricted sensitivity analysis because of limited observed HRQoL data [49]. Their inclusion in the primary analysis increased reliance on imputation. Nevertheless, attrition may have introduced some selection bias. Although the 547 participants included in the sensitivity analysis and the 106 additional ITT-SHI participants were largely comparable with respect to baseline demographic and clinical characteristics, the additional participants were more often male and more likely to have three or more children. These findings suggest that missingness was associated with some observed baseline characteristics and therefore do not support a missing-completely-at-random (MCAR) mechanism. However, the sensitivity analysis in the restricted population yielded results consistent with the primary analysis for CF and WC, whereas mean costs and cost-effectiveness estimates for CF + WC differed between the populations. Although the observed data were consistent with missing at random (MAR), a missing not at random (MNAR) mechanism cannot be ruled out based on the observed data alone.

For missing HRQoL at T4, multiple imputation was used in 220 cases (33.7%), and costs at T3-T4 were imputed in 242 instances (37.1%). Although multiple imputation is an appropriate approach for handling missing data, the relatively high proportion of imputed values may have introduced uncertainty into the estimates. To assess the robustness of our findings, we conducted a sensitivity analysis using a restricted analysis population (*n* = 547) as described in the Methods section. The resulting cost-effectiveness estimates, including CEACs and rank probabilities, were broadly consistent with the primary analysis for CF and WC, whereas the findings for CF + WC differed. Thus, the results for CF + WC were sensitive to the definition of the analysis population. Cost data often show skewed distributions in health economic research, where most individuals incur relatively low costs, but some experience very high expenses [50, 51]. The leave-one-out analyses additionally demonstrated sensitivity to individual extreme cost observations. These analyses do not resolve uncertainty arising from potentially informative missingness.

The exclusion of individuals from non-participating SHI companies (*n* = 512) was unrelated to intervention assignment. However, comparison of EQ-5D scores between individuals insured with participating versus non-participating SHI funds revealed small differences across the intervention and control groups, which may affect the external validity of the study results (Supplementary Table 9). Moreover, WC participation was restricted to one family-selected parent per family, and the selection of this parent was not randomized, complicating direct comparisons between WC participants and CG counterparts. Furthermore, the study’s design confined the evaluation of HRQoL to an 18-month period post-enrollment. Extending the follow-up period over several years with a larger sample size would be instrumental in understanding the enduring effects on HRQoL.

## Conclusions

In the primary analysis, CF and WC were associated with lower mean costs and positive baseline-adjusted QALY differences relative to the control group, whereas CF + WC was associated with higher mean costs and a positive baseline-adjusted QALY difference. None of the differences in costs or baseline-adjusted QALYs were statistically significant. At a WTP of €50,000 per QALY, the probabilities of being cost-effective compared with CG were 84.2% for CF, 79.9% for WC, and 43.0% for CF + WC. The sensitivity analysis using the restricted population yielded findings consistent with the primary analysis for CF and WC, whereas CF + WC was associated with lower mean costs than the control group. Leave-one-out analyses demonstrated that cost-effectiveness estimates were sensitive to individual extreme cost observations, particularly for CF + WC. Differences in costs and baseline-adjusted QALYs remained statistically non-significant across all intervention groups in the sensitivity analysis. Given the study limitations, particularly the relatively small sample size, the current evidence is insufficient to support a definitive recommendation for implementation into routine care from a health economic perspective. Further research with larger study populations and longer follow-up is recommended to robustly substantiate the long-term cost-effectiveness and benefits of these interventions.

Ultimately, the incorporation of such interventions into the healthcare system relies on the decisions of policymakers, who must weigh these findings alongside other clinical and societal considerations.

## Supplementary Information

Supplementary Material 1.

Supplementary Material 2.

Supplementary Material 3.

## Data Availability

Participants provided written informed consent under the condition of confidentiality of their data including restricted access of third parties. Therefore, data cannot be shared.
