# Supplementary material for: Families caring for children and adolescents with rare disease: a health economic evaluation of face-to-face and online psychosocial support intervention of the randomized controlled CARE-FAM-NET study
Source: Health Econ Rev. 2026 Oct 26;16:129. doi: 10.1186/s13561-026-00883-0 (PMC13721576; doi:10.1186/s13561-026-00883-0)
Supplement: Supplementary file 2 [file 13561_2026_883_MOESM2_ESM.docx]

**Supplementary Figure 1: Cost-effectiveness planes of interventions CF, WC and CF+WC compared with CG (n = 547)**

**Supplementary Figure 2: Cost-effectiveness acceptability curves for interventions CF, WC and CF+WC compared to the CG (n = 547)**

**Supplementary Figure 3: Probability of being the most cost-effective option across willingness-to-pay thresholds (WTP) (sensitivity population, n = 547)**

**Supplementary Figure 4: Rankogram at a WTP of €50000 per QALY (sensitivity population, n = 547)**
