# Supplementary material for: Families caring for children and adolescents with rare disease: a health economic evaluation of face-to-face and online psychosocial support intervention of the randomized controlled CARE-FAM-NET study
Source: Health Econ Rev. 2026 Oct 26;16:129. doi: 10.1186/s13561-026-00883-0 (PMC13721576; doi:10.1186/s13561-026-00883-0)
Supplement: Supplementary file 3 [file 13561_2026_883_MOESM3_ESM.docx]

**Supplementary Table 2: Baseline characteristics of the sensitivity analysis population and additional participants included in the ITT-SHI population**

| **Characteristic** | **Sensitivity analysis participants** | **Additional ITT-SHI participants** | **p-value** |
| --- | --- | --- | --- |
| **Sex** |  |  | 0.014 |
| Female [n %] | 343 (62.7%) | 53 (50.0%) |  |
| Male [n %] | 204 (37.3%) | 53 (50.0%) |  |
| **Age, mean (SD)** | 39.42 (7.22) | 39.44 (7.34) | 0.978 |
| **Education** |  |  | 0.489 |
| Low^a^ [n (%)] | 47 (9.2%) | 6 (6.1%) |  |
| Moderate^a^ [n (%)] | 159 (31.1%) | 35 (35.4%) |  |
| High^a^ [n (%)] | 306 (59.8%) | 58 (58.6%) |  |
| **Marital status or with a partner** |  |  | 0.559 |
| Married/partnered | 446 (82.0%) | 90 (84.9%) |  |
| Not married/partnered | 98 (18.0%) | 16 (15.1%) |  |
| **Number of children** |  |  | 0.002 |
| 1 | 165 (30.2%) | 14 (13.3%) |  |
| 2 | 285 (52.2%) | 61 (58.1%) |  |
| 3 | 66 (12.1%) | 21 (20.0%) |  |
| 4+ | 30 (5.5%) | 9 (8.6%) |  |
| **Suffering from mental illness [n (%)]** |  |  | 0.694 |
| No | 484 (90.3%) | 92 (88.5%) |  |
| Yes | 52 (9.7%) | 12 (11.5%) |  |
| **BSI Global Severity Index, mean (SD)** | 0.37 (0.37) | 0.43 (0.42) | 0.143 |
| **BSI Anxiety subscale score, mean (SD)** | 0.31 (0.40) | 0.38 (0.40) | 0.113 |
| **EQ-5D-3L-Score (T1), mean (SD)** | 0.92 (0.14) | 0.91 (0.14) | 0.468 |

Continuous variables are presented as mean (standard deviation, SD). Categorical variables are presented as number (percentage, %). P-values are based on t-tests for continuous variables and chi-square tests for categorical variables. ^a^Low refers to 9 years of schooling, moderate to 10 years, and high to 12–13 years.

**Supplementary Table 3: Dimension specific EQ-5D-3L values**

| **EQ-5D-3L dimension** | **Response level** | **Study group** | **T1** | **T2** | **T3** | **T4** |
| --- | --- | --- | --- | --- | --- | --- |
| Usual activities | Level 1 | CG | 128 (89.5%) | 116 (91.3%) | 103 (85.8%) | 102 (86.4%) |
| Usual activities | Level 1 | CF | 122 (86.5%) | 123 (91.8%) | 102 (86.4%) | 105 (89.0%) |
| Usual activities | Level 1 | WC | 121 (89.6%) | 101 (84.9%) | 100 (89.3%) | 94 (89.5%) |
| Usual activities | Level 1 | CF+WC | 110 (85.9%) | 107 (89.2%) | 86 (87.8%) | 81 (87.1%) |
| Usual activities | Level 2 | CG | 15 (10.5%) | 10 (7.9%) | 16 (13.3%) | 15 (12.7%) |
| Usual activities | Level 2 | CF | 19 (13.5%) | 11 (8.2%) | 16 (13.6%) | 12 (10.2%) |
| Usual activities | Level 2 | WC | 14 (10.4%) | 18 (15.1%) | 12 (10.7%) | 10 (9.5%) |
| Usual activities | Level 2 | CF+WC | 18 (14.1%) | 13 (10.8%) | 12 (12.2%) | 12 (12.9%) |
| Usual activities | Level 3 | CG | 0 (0.0%) | 1 (0.8%) | 1 (0.8%) | 1 (0.8%) |
| Usual activities | Level 3 | CF | 0 (0.0%) | 0 (0.0%) | 0 (0.0%) | 1 (0.8%) |
| Usual activities | Level 3 | WC | 0 (0.0%) | 0 (0.0%) | 0 (0.0%) | 1 (1.0%) |
| Mobility | Level 1 | CG | 133 (93.0%) | 114 (89.8%) | 110 (91.7%) | 101 (85.6%) |
| Mobility | Level 1 | CF | 127 (90.1%) | 120 (90.2%) | 104 (88.1%) | 103 (87.3%) |
| Mobility | Level 1 | WC | 124 (91.9%) | 113 (95.0%) | 104 (92.9%) | 97 (92.4%) |
| Mobility | Level 1 | CF+WC | 119 (93.0%) | 112 (93.3%) | 87 (88.8%) | 85 (91.4%) |
| Mobility | Level 2 | CG | 10 (7.0%) | 13 (10.2%) | 10 (8.3%) | 17 (14.4%) |
| Mobility | Level 2 | CF | 14 (9.9%) | 13 (9.8%) | 14 (11.9%) | 15 (12.7%) |
| Mobility | Level 2 | WC | 11 (8.1%) | 6 (5.0%) | 8 (7.1%) | 8 (7.6%) |
| Mobility | Level 2 | CF+WC | 9 (7.0%) | 8 (6.7%) | 11 (11.2%) | 8 (8.6%) |
| Pain/discomfort | Level 1 | CG | 77 (53.8%) | 63 (49.6%) | 64 (53.3%) | 61 (51.7%) |
| Pain/discomfort | Level 1 | CF | 87 (61.7%) | 74 (55.2%) | 70 (59.8%) | 75 (64.1%) |
| Pain/discomfort | Level 1 | WC | 85 (63.0%) | 72 (61.0%) | 71 (63.4%) | 65 (61.9%) |
| Pain/discomfort | Level 1 | CF+WC | 72 (56.2%) | 73 (60.8%) | 60 (61.2%) | 60 (64.5%) |
| Pain/discomfort | Level 2 | CG | 61 (42.7%) | 61 (48.0%) | 55 (45.8%) | 55 (46.6%) |
| Pain/discomfort | Level 2 | CF | 49 (34.8%) | 56 (41.8%) | 43 (36.8%) | 39 (33.3%) |
| Pain/discomfort | Level 2 | WC | 46 (34.1%) | 45 (38.1%) | 40 (35.7%) | 38 (36.2%) |
| Pain/discomfort | Level 2 | CF+WC | 54 (42.2%) | 47 (39.2%) | 37 (37.8%) | 31 (33.3%) |
| Pain/discomfort | Level 3 | CG | 5 (3.5%) | 3 (2.4%) | 1 (0.8%) | 2 (1.7%) |
| Pain/discomfort | Level 3 | CF | 5 (3.5%) | 4 (3.0%) | 4 (3.4%) | 3 (2.6%) |
| Pain/discomfort | Level 3 | WC | 4 (3.0%) | 1 (0.8%) | 1 (0.9%) | 2 (1.9%) |
| Pain/discomfort | Level 3 | CF+WC | 2 (1.6%) | 0 (0.0%) | 1 (1.0%) | 2 (2.2%) |
| Self-care | Level 1 | CG | 142 (99.3%) | 124 (97.6%) | 116 (96.7%) | 116 (98.3%) |
| Self-care | Level 1 | CF | 137 (97.2%) | 129 (96.3%) | 114 (96.6%) | 113 (95.8%) |
| Self-care | Level 1 | WC | 133 (98.5%) | 118 (99.2%) | 112 (100.0%) | 101 (96.2%) |
| Self-care | Level 1 | CF+WC | 126 (98.4%) | 117 (97.5%) | 94 (95.9%) | 89 (95.7%) |
| Self-care | Level 2 | CG | 1 (0.7%) | 3 (2.4%) | 4 (3.3%) | 2 (1.7%) |
| Self-care | Level 2 | CF | 4 (2.8%) | 5 (3.7%) | 4 (3.4%) | 5 (4.2%) |
| Self-care | Level 2 | WC | 2 (1.5%) | 1 (0.8%) | 0 (0.0%) | 4 (3.8%) |
| Self-care | Level 2 | CF+WC | 2 (1.6%) | 3 (2.5%) | 4 (4.1%) | 4 (4.3%) |
| Anxiety/depression | Level 1 | CG | 92 (64.3%) | 84 (66.1%) | 83 (69.2%) | 81 (68.6%) |
| Anxiety/depression | Level 1 | CF | 71 (51.1%) | 92 (69.2%) | 94 (79.7%) | 89 (75.4%) |
| Anxiety/depression | Level 1 | WC | 86 (63.7%) | 82 (68.9%) | 83 (74.1%) | 81 (77.1%) |
| Anxiety/depression | Level 1 | CF+WC | 83 (64.8%) | 79 (65.8%) | 68 (69.4%) | 68 (73.1%) |
| Anxiety/depression | Level 2 | CG | 49 (34.3%) | 42 (33.1%) | 36 (30.0%) | 36 (30.5%) |
| Anxiety/depression | Level 2 | CF | 67 (48.2%) | 41 (30.8%) | 24 (20.3%) | 28 (23.7%) |
| Anxiety/depression | Level 2 | WC | 43 (31.9%) | 32 (26.9%) | 28 (25.0%) | 24 (22.9%) |
| Anxiety/depression | Level 2 | CF+WC | 42 (32.8%) | 39 (32.5%) | 29 (29.6%) | 22 (23.7%) |
| Anxiety/depression | Level 3 | CG | 2 (1.4%) | 1 (0.8%) | 1 (0.8%) | 1 (0.8%) |
| Anxiety/depression | Level 3 | CF | 1 (0.7%) | 0 (0.0%) | 0 (0.0%) | 1 (0.8%) |
| Anxiety/depression | Level 3 | WC | 6 (4.4%) | 5 (4.2%) | 1 (0.9%) | 0 (0.0%) |
| Anxiety/depression | Level 3 | CF+WC | 3 (2.3%) | 2 (1.7%) | 1 (1.0%) | 3 (3.2%) |

Values are presented as n (%). Percentages are based on participants with available data for the respective EQ-5D-3L dimension, study group, and assessment time point. Level 1 = no problems; Level 2 = some problems; Level 3 = extreme problems. T1–T4 = assessment time points.

**Supplementary Table 4: Cost by healthcare sector in the ITT-SHI population (n = 653)**

| **Cost category** | **CG** | **CF** | **WC** | **CF+WC** | **All participants** |
| --- | --- | --- | --- | --- | --- |
| Outpatient care | 2,624 (4,267) | 1,924 (5,282) | 1,978 (2,772) | 2,515 (5,257) | 2,252 (4,463) |
| Medication | 851 (4,799) | 1,079 (8,712) | 2,047 (23,251) | 3,188 (30,622) | 1,751 (19,453) |
| Rehabilitation | 215 (1,103) | 53 (476) | 44 (410) | 57 (512) | 93 (691) |
| Outpatient long-term nursing care | 52 (148) | 80 (474) | 167 (1,346) | 122 (382) | 105 (761) |
| Remedies (Heilmittel) | 144 (515) | 250 (1,002) | 137 (508) | 246 (702) | 192 (707) |
| Hospital (inpatient) care | 761 (2,951) | 498 (1,954) | 523 (1,861) | 560 (1,761) | 587 (2,195) |
| Sick pay / incapacity for work | 664 (2,938) | 502 (1,636) | 534 (2,588) | 368 (2,849) | 522 (2,548) |
| Intervention costs | 0 (0) | 234 (0) | 140 (185) | 377 (183) | 180 (187) |
| **Total costs** | **5,311 (10,211)** | **4,620 (13,767)** | **5,570 (24,916)** | **7,433 (33,150)** | **5,682 (22,023)** |

Values shown as mean (SD) in EUR, summed over the 18-month analysis period (T1-T4), based on observed (non-imputed) sector-level data, ITT-SHI population (n=653). Total costs include healthcare costs and intervention costs.

**Supplementary Table 5: Cost by healthcare sector in the sensitivity analysis population (n = 547)**

| **Cost category** | **CG** | **CF** | **WC** | **CF+WC** | **All participants** |
| --- | --- | --- | --- | --- | --- |
| Outpatient care | 2,631 (4,434) | 2,054 (5,631) | 1,971 (2,551) | 2,638 (5,571) | 2,321 (4,707) |
| Medication | 896 (5,192) | 1,231 (9,361) | 2,523 (26,464) | 742 (3,884) | 1,348 (14,330) |
| Rehabilitation | 254 (1,196) | 28 (318) | 57 (466) | 65 (546) | 103 (727) |
| Outpatient long-term nursing care | 49 (137) | 92 (509) | 187 (1,519) | 111 (342) | 109 (817) |
| Remedies (Heilmittel) | 162 (557) | 283 (1,074) | 122 (471) | 250 (737) | 204 (749) |
| Hospital (inpatient) care | 733 (2,971) | 497 (2,001) | 403 (1,480) | 510 (1,441) | 539 (2,087) |
| Sick pay / incapacity for work | 634 (2,781) | 542 (1,748) | 631 (2,896) | 413 (3,041) | 558 (2,648) |
| Intervention costs | 0 (0) | 234 (0) | 159 (192) | 388 (187) | 190 (191) |
| **Total costs** | **5,360 (10,491)** | **4,961 (14,729)** | **6,052 (28,246)** | **5,117 (9,292)** | **5,371 (17,330)** |

Values shown as mean (SD) in EUR, summed over the 18-month analysis period (T1-T4). Sector-level costs are based on observed (non-imputed) data. Total costs represent the sum of the sector-level costs and intervention costs.

**Supplementary Table 6: Comparison between imputed and observed EQ-5D-3L scores (n = 653)**

|  |  | **Imputed results** | | | | | | | | **Observed results** | | | | | | | | |
| --- | --- | --- | --- | --- | --- | --- | --- | --- | --- | --- | --- | --- | --- | --- | --- | --- | --- | --- |
| **Group** | **Variable** | **N** | **Mean** | **SD** | **Median** | **Q1** | **Q3** | **Min** | **Max** | **N** | **Mean** | **SD** | **Median** | **Q1** | **Q3** | **Min** | **Max** |  |
| CG | T1 Score | 169 | 0.9144 | 0.1492 | 0.9990 | 0.887 | 1 | 0.175 | 1 | 169 | 0.9144 | 0.1492 | 0.9990 | 0.887 | 1 | 0.175 | 1 |  |
|  | T2 Score | 169 | 0.9172 | 0.1305 | 0.9598 | 0.887 | 1 | 0.197 | 1 | 127 | 0.9153 | 0.1327 | 0.8870 | 0.887 | 1 | 0.197 | 1 |  |
|  | T3 Score | 169 | 0.9221 | 0.1217 | 0.9780 | 0.887 | 1 | 0.175 | 1 | 120 | 0.9257 | 0.1149 | 0.9990 | 0.887 | 1 | 0.175 | 1 |  |
|  | T4 Score | 169 | 0.9202 | 0.1217 | 0.9934 | 0.887 | 1 | 0.262 | 1 | 118 | 0.9146 | 0.1267 | 0.9990 | 0.887 | 1 | 0.262 | 1 |  |
| CF | T1 Score | 163 | 0.9207 | 0.1571 | 0.9990 | 0.887 | 1 | 0.175 | 1 | 161 | 0.9212 | 0.1569 | 0.9990 | 0.887 | 1 | 0.175 | 1 |  |
|  | T2 Score | 163 | 0.9228 | 0.1337 | 0.9853 | 0.887 | 1 | 0.175 | 1 | 132 | 0.9195 | 0.1421 | 0.9990 | 0.887 | 1 | 0.175 | 1 |  |
|  | T3 Score | 163 | 0.9292 | 0.1301 | 1.0000 | 0.887 | 1 | 0.175 | 1 | 117 | 0.9217 | 0.1454 | 1.0000 | 0.887 | 1 | 0.175 | 1 |  |
|  | T4 Score | 163 | 0.9318 | 0.1385 | 1.0000 | 0.887 | 1 | 0.110 | 1 | 117 | 0.9261 | 0.1529 | 1.0000 | 0.887 | 1 | 0.110 | 1 |  |
| WC | T1 Score | 175 | 0.9164 | 0.1489 | 0.9990 | 0.887 | 1 | 0.197 | 1 | 175 | 0.9164 | 0.1489 | 0.9990 | 0.887 | 1 | 0.197 | 1 |  |
|  | T2 Score | 175 | 0.9281 | 0.1167 | 0.9990 | 0.887 | 1 | 0.296 | 1 | 118 | 0.9319 | 0.1167 | 0.9990 | 0.887 | 1 | 0.296 | 1 |  |
|  | T3 Score | 175 | 0.9457 | 0.0994 | 0.9995 | 0.887 | 1 | 0.262 | 1 | 112 | 0.9433 | 0.1055 | 0.9995 | 0.887 | 1 | 0.262 | 1 |  |
|  | T4 Score | 175 | 0.9358 | 0.1185 | 0.9997 | 0.887 | 1 | 0.175 | 1 | 105 | 0.9329 | 0.1286 | 1.0000 | 0.887 | 1 | 0.175 | 1 |  |
| CF+WC | T1 Score | 146 | 0.9286 | 0.1113 | 0.9990 | 0.887 | 1 | 0.296 | 1 | 146 | 0.9286 | 0.1113 | 0.9990 | 0.887 | 1 | 0.296 | 1 |  |
|  | T2 Score | 146 | 0.9385 | 0.0929 | 0.9990 | 0.887 | 1 | 0.499 | 1 | 120 | 0.9403 | 0.0887 | 0.9990 | 0.887 | 1 | 0.499 | 1 |  |
|  | T3 Score | 146 | 0.9369 | 0.1095 | 0.9994 | 0.887 | 1 | 0.110 | 1 | 98 | 0.9354 | 0.1126 | 0.9990 | 0.887 | 1 | 0.110 | 1 |  |
|  | T4 Score | 146 | 0.9360 | 0.1310 | 0.9999 | 0.887 | 1 | 0.110 | 1 | 93 | 0.9272 | 0.1445 | 1.0000 | 0.887 | 1 | 0.110 | 1 |  |

Imputed results are based on 20 multiply imputed datasets. Observed results are based on available non-imputed EQ-5D-3L values. N indicates the number of participants contributing to the respective analysis.

|  |  | **Imputed results** | | | | | | | | | **Observed results** | | | | | | | | |
| --- | --- | --- | --- | --- | --- | --- | --- | --- | --- | --- | --- | --- | --- | --- | --- | --- | --- | --- | --- |
| **Group** | **Variable** | **N** | **Mean** | **SD** | **Median** | **Q1** | **Q3** | **Min** | **Max** | **N** | | **Mean** | **SD** | **Median** | **Q1** | **Q3** | **Min** | **Max** |  |
| CG | Total Costs T0-T1 | 169 | 3,841 | 5,853 | 1,473 | 412 | 5,081 | 0 | 31,542 | 157 | | 3,725 | 5,753 | 1,401 | 401 | 4,992 | 0 | 31,542 |  |
|  | Total Costs T1-T2 | 169 | 2,686 | 5,103 | 691 | 226 | 2,587 | 0 | 32,594 | 161 | | 2,603 | 5,008 | 672 | 218 | 2,530 | 0 | 32,594 |  |
|  | Total Costs T2-T3 | 169 | 1,894 | 4,623 | 387 | 87 | 1,583 | 0 | 33,616 | 144 | | 1,835 | 4,632 | 384 | 79 | 1,513 | 0 | 33,616 |  |
|  | Total Costs T3-T4 | 169 | 2,165 | 4,476 | 409 | 67 | 1,736 | 0 | 31,519 | 106 | | 2,020 | 4,414 | 390 | 63 | 1,450 | 0 | 31,519 |  |
| CF | Total Costs T0-T1 | 163 | 3,247 | 4,966 | 1,281 | 367 | 4,609 | 0 | 33,630 | 150 | | 3,350 | 5,090 | 1,346 | 380 | 4,659 | 0 | 33,630 |  |
|  | Total Costs T1-T2 | 163 | 2,584 | 8,363 | 531 | 135 | 1,706 | 0 | 78,218 | 155 | | 2,628 | 8,520 | 537 | 133 | 1,705 | 0 | 78,218 |  |
|  | Total Costs T2-T3 | 163 | 1,483 | 4,451 | 279 | 72 | 815 | 0 | 39,318 | 136 | | 1,443 | 4,460 | 256 | 64 | 803 | 0 | 39,318 |  |
|  | Total Costs T3-T4 | 163 | 1,022 | 3,003 | 178 | 44 | 647 | 0 | 31,325 | 97 | | 1,145 | 3,602 | 179 | 43 | 647 | 0 | 31,325 |  |
| WC | Total Costs T0-T1 | 175 | 3,905 | 12,323 | 1,160 | 313 | 3,855 | 0 | 149,076 | 172 | | 3,915 | 12,404 | 1,155 | 316 | 3,864 | 0 | 149,076 |  |
|  | Total Costs T1-T2 | 175 | 2,380 | 9,762 | 742 | 169 | 2,071 | 0 | 124,859 | 174 | | 2,388 | 9,789 | 743 | 168 | 2,073 | 0 | 124,859 |  |
|  | Total Costs T2-T3 | 175 | 1,922 | 8,544 | 472 | 42 | 1,494 | 0 | 108,704 | 156 | | 2,054 | 9,026 | 482 | 46 | 1,573 | 0 | 108,704 |  |
|  | Total Costs T3-T4 | 175 | 1,536 | 7,136 | 517 | 45 | 1,094 | 0 | 92,163 | 116 | | 1,849 | 8,712 | 522 | 40 | 1,120 | 0 | 92,163 |  |
| CF+WC | Total Costs T0-T1 | 146 | 5,423 | 27,023 | 1,326 | 573 | 3,855 | 0 | 323,563 | 144 | | 5,470 | 27,207 | 1,330 | 574 | 3,878 | 0 | 323,563 |  |
|  | Total Costs T1-T2 | 146 | 3,737 | 18,687 | 574 | 154 | 1,867 | 0 | 218,923 | 144 | | 3,765 | 18,812 | 572 | 153 | 1,884 | 0 | 218,923 |  |
|  | Total Costs T2-T3 | 146 | 2,556 | 13,000 | 314 | 67 | 1,401 | 0 | 152,947 | 126 | | 2,694 | 13,905 | 354 | 67 | 1,434 | 0 | 152,947 |  |
|  | Total Costs T3-T4 | 146 | 1,631 | 4,373 | 328 | 49 | 1,373 | 0 | 29,336 | 92 | | 1,615 | 4,385 | 305 | 42 | 1,364 | 0 | 29,336 |  |

**Supplementary Table 7: Comparison between imputed and observed costs (n = 653)**

Costs are presented in euros. Observed and imputed costs refer to SHI-covered healthcare costs. Imputed results are based on 20 multiply imputed datasets. Observed results are based on available non-imputed cost data. N indicates the number of participants contributing to the respective analysis.

**Supplementary Table 8: Mean Costs, mean QALYs and Incremental Values (n = 547)**

|  | **CG (n=143)** | **CF (n=141)** | **WC (n=135)** | **CF+WC (n=128)** |
| --- | --- | --- | --- | --- |
| Mean total Costs (SE)^a^ | €6804 (€1094) | €5639 (€1292) | €6372 (€2419) | €6098 (€1003) |
| GLM Cost ratio (95% CI)^b^ ; *p*-value | Reference | 0.8300 (0.4754-1.4489); *p=0.5120* | 0.9382 (0.4185-2.1034); *p=0.8770* | 0.8964 (0.5559-1.4454); *p=0.6531* |
| ∆ Mean total Costs | Reference | -€1165 | -€432 | -€706 |
| Mean annual Costs (SE)^d^ | €4536 (€729) | €3759 (€861) | €4248 (€1613) | €4065 (€669) |
| ∆Mean annual Costs ^d^ | Reference | -€777 | -€288 | -€471 |
| Mean total QALYs (SE)^a^ | 1.3729 (0.0123) | 1.3787 (0.0163) | 1.3916 (0.0132) | 1.3964 (0.0115) |
| Adjusted mean difference in total QALYs (95% CI); *p*-value *^c^* | Reference | 0.0029 (-0.0221-0.0279); *p=0.8211* | 0.0190 (-0.0031-0.0410); *p=0.0916* | 0.0163 (-0.0092-0.0418); *p=0.2108* |
| ∆ Mean total QALYs | Reference | 0.0058 | 0.0187 | 0.0235 |
| Mean annual QALYs (SE)^d^ | 0.9153 (0.0082) | 0.9191 (0.0109) | 0.9277 (0.0088) | 0.9309 (0.0077) |
| ∆Mean annual QALYs ^d^ | Reference | 0.0039 | 0.0125 | 0.0157 |
| ICER vs. CG (∆Cost/∆QALY)^e^ | Reference | Dominates CG | Dominates CG | Dominates CG |

CF: Intervention CARE-FAM, CF+WC: Intervention CARE-FAM+WEP-CARE, CG: Control group, QALY: Quality-Adjusted Life Year, SE: Standard error, WC: Intervention WEP-CARE, ^a^Unadjusted mean total discounted costs including intervention costs and unadjusted mean discounted QALYs per parent during the 18-month observation period (T1 to T4), ^b^Cost ratios derived from a GLM (log link) and family-cluster-robust standard errors, pooled across m = 20 MI-datasets using Rubin’s Rules. ^c^Baseline-adjusted mean differences in total discounted QALYs relative to CG were estimated using linear regression with treatment group and baseline EQ-5D-3L utility as explanatory variables and family-clustered CR2 robust standard errors. Estimates were pooled across 20 MI-datasets using Rubin’s Rules. ^d^Mean unadjusted annual discounted costs or QALYs per parent (T1 to T4); annualized values assume uniform costs over the 18-month observation period. ^e^ICERs were calculated by dividing the difference in pooled unadjusted mean total discounted costs by the baseline-adjusted mean difference in total discounted QALYs.

**Supplementary Table 9: Comparison of EQ-5D scores between individuals insured with participating vs. non-participating SHI**

| **Group** | **Insured with participating SHI** | **EQ-5D-3L Score** | **N** | **Mean** | **SD** | **Median** | **Q1** | **Q3** | **Min** | **Max** |
| --- | --- | --- | --- | --- | --- | --- | --- | --- | --- | --- |
| CG | Yes | T1 | 143 | 0,9186 | 0,1469 | 0,9990 | 0,8870 | 1 | 0,1750 | 1 |
|  |  | T2 | 127 | 0,9153 | 0,1327 | 0,8870 | 0,8870 | 1 | 0,1970 | 1 |
|  |  | T3 | 120 | 0,9257 | 0,1149 | 0,9990 | 0,8870 | 1 | 0,1750 | 1 |
|  |  | T4 | 118 | 0,9146 | 0,1267 | 0,9990 | 0,8870 | 1 | 0,2620 | 1 |
|  | No | T1 | 82 | 0,9325 | 0,1217 | 0,9990 | 0,8870 | 1 | 0,2620 | 1 |
|  |  | T2 | 71 | 0,9608 | 0,0633 | 1,0000 | 0,8870 | 1 | 0,7010 | 1 |
|  |  | T3 | 56 | 0,9333 | 0,0758 | 0,9990 | 0,8870 | 1 | 0,7880 | 1 |
|  |  | T4 | 53 | 0,9330 | 0,0717 | 0,8870 | 0,8870 | 1 | 0,7880 | 1 |
| CF | Yes | T1 | 141 | 0,9225 | 0,1575 | 0,9990 | 0,8870 | 1 | 0,1750 | 1 |
|  |  | T2 | 132 | 0,9195 | 0,1421 | 0,9990 | 0,8870 | 1 | 0,1750 | 1 |
|  |  | T3 | 117 | 0,9217 | 0,1454 | 1,0000 | 0,8870 | 1 | 0,1750 | 1 |
|  |  | T4 | 117 | 0,9261 | 0,1529 | 1,0000 | 0,8870 | 1 | 0,1100 | 1 |
|  | No | T1 | 95 | 0,9038 | 0,1603 | 0,9990 | 0,8870 | 1 | 0,1750 | 1 |
|  |  | T2 | 88 | 0,9353 | 0,0948 | 1,0000 | 0,8870 | 1 | 0,4030 | 1 |
|  |  | T3 | 73 | 0,9101 | 0,1538 | 0,8870 | 0,8870 | 1 | -0,0550 | 1 |
|  |  | T4 | 72 | 0,9205 | 0,1112 | 0,9995 | 0,8870 | 1 | 0,4990 | 1 |
| WC | Yes | T1 | 135 | 0,9182 | 0,1541 | 0,9990 | 0,8870 | 1 | 0,1970 | 1 |
|  |  | T2 | 118 | 0,9319 | 0,1167 | 0,9990 | 0,8870 | 1 | 0,2960 | 1 |
|  |  | T3 | 112 | 0,9433 | 0,1055 | 0,9995 | 0,8870 | 1 | 0,2620 | 1 |
|  |  | T4 | 105 | 0,9329 | 0,1286 | 1,0000 | 0,8870 | 1 | 0,1750 | 1 |
|  | No | T1 | 86 | 0,9342 | 0,0834 | 0,9990 | 0,8870 | 1 | 0,6110 | 1 |
|  |  | T2 | 78 | 0,9146 | 0,1345 | 0,8870 | 0,8870 | 1 | 0,1750 | 1 |
|  |  | T3 | 65 | 0,9286 | 0,1436 | 1,0000 | 0,8870 | 1 | 0,1750 | 1 |
|  |  | T4 | 66 | 0,9325 | 0,1109 | 0,9990 | 0,8870 | 1 | 0,2620 | 1 |
| CF+WC | Yes | T1 | 128 | 0,9273 | 0,1171 | 0,9990 | 0,8870 | 1 | 0,2960 | 1 |
|  |  | T2 | 120 | 0,9403 | 0,0887 | 0,9990 | 0,8870 | 1 | 0,4990 | 1 |
|  |  | T3 | 98 | 0,9354 | 0,1126 | 0,9990 | 0,8870 | 1 | 0,1100 | 1 |
|  |  | T4 | 93 | 0,9272 | 0,1445 | 1,0000 | 0,8870 | 1 | 0,1100 | 1 |
|  | No | T1 | 96 | 0,9339 | 0,1176 | 0,9990 | 0,8870 | 1 | 0,1870 | 1 |
|  |  | T2 | 90 | 0,9250 | 0,1453 | 0,9990 | 0,8870 | 1 | 0,1750 | 1 |
|  |  | T3 | 70 | 0,9304 | 0,1156 | 0,9990 | 0,8870 | 1 | 0,1970 | 1 |
|  |  | T4 | 68 | 0,9298 | 0,1148 | 0,9995 | 0,8870 | 1 | 0,1970 | 1 |
